# Gut-derived short-chain fatty acids modulate skin barrier integrity by promoting keratinocyte metabolism and differentiation

**DOI:** 10.1038/s41385-022-00524-9

**Published:** 2022-06-07

**Authors:** Aurélien Trompette, Julie Pernot, Olaf Perdijk, Rayed Ali A. Alqahtani, Jaime Santo Domingo, Dolores Camacho-Muñoz, Nicholas C. Wong, Alexandra C. Kendall, Andreas Wiederkehr, Laurent P. Nicod, Anna Nicolaou, Christophe von Garnier, Niki D. J. Ubags, Benjamin J. Marsland

**Affiliations:** 1 Division of Pulmonary Medicine, Department of Medicine, Lausanne University Hospital (CHUV), University of Lausanne (UNIL), Lausanne, Switzerland; 2 Department of Immunology and Pathology, Central Clinical School, Monash University, Melbourne, VIC Australia; 3 Laboratory for Lipidomics and Lipid Biology, University of Manchester, Division of Pharmacy and Optometry, Faculty of Biology Medicine and Health, Manchester Academic Health Science Centre, The University of Manchester, Manchester, M13 9PT UK; 4 Nestlé Institute of Health, EPFL innovation Park, Lausanne, Switzerland; 5 Monash Bioinformatics Platform, Monash University, Clayton, VIC Australia; 6 Pneumologie, Clinic Cecil from Hirslanden, Lausanne, Switzerland

## Abstract

Barrier integrity is central to the maintenance of healthy immunological homeostasis. Impaired skin barrier function is linked with enhanced allergen sensitization and the development of diseases such as atopic dermatitis (AD), which can precede the development of other allergic disorders, for example, food allergies and asthma. Epidemiological evidence indicates that children suffering from allergies have lower levels of dietary fibre-derived short-chain fatty acids (SCFA). Using an experimental model of AD-like skin inflammation, we report that a fermentable fibre-rich diet alleviates systemic allergen sensitization and disease severity. The gut-skin axis underpins this phenomenon through SCFA production, particularly butyrate, which strengthens skin barrier function by altering mitochondrial metabolism of epidermal keratinocytes and the production of key structural components. Our results demonstrate that dietary fibre and SCFA improve epidermal barrier integrity, ultimately limiting early allergen sensitization and disease development.

The Graphical Abstract was designed using Servier Medical Art images (https://smart.servier.com).
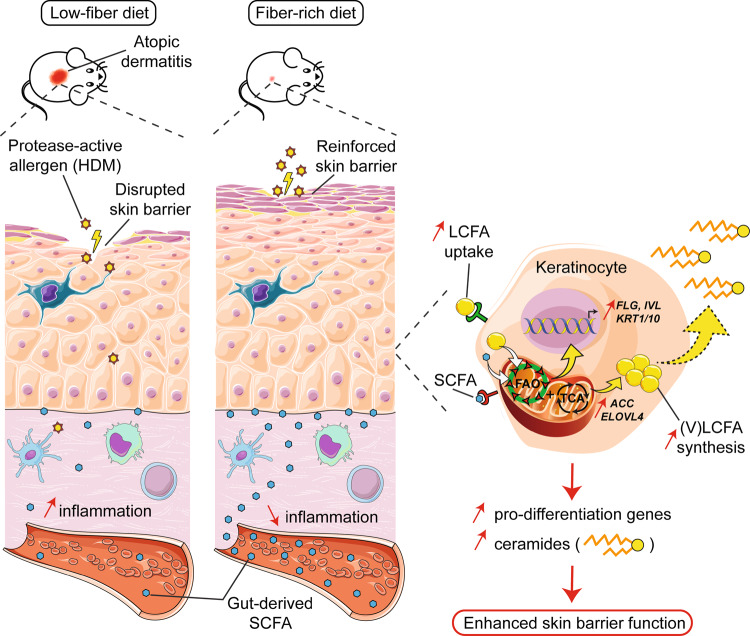

## Introduction

Over the past decades, the incidence of chronic inflammatory disorders such as autoimmune and type 2 allergic diseases has increased worldwide^1–4^. Allergic disorders alone affect nearly one-third of the global population, representing a substantial social and economic burden^5,6^. A growing body of evidence indicates that this rapid and relatively recent onset of allergic epidemics is the consequence of environmental and behavioral changes collectively known as the “westernized lifestyle”^7,8^. More recently, it was hypothesized that such a lifestyle could cause epithelial barrier dysfunction, an event leading to enhanced ingress of allergens at mucosal surfaces, systemic allergen sensitization, and ultimately the development of allergic diseases^9,10^.

Atopic dermatitis (AD), a chronic and relapsing inflammatory skin disorder, manifests in the first years of life and affects ~20% of children worldwide^11^. Data from epidemiological studies suggest that allergic disorders develop in a timely and orderly manner, from AD and food allergy in infants to allergic rhinitis and asthma later in life^12–14^, a phenomenon referred to as the “atopic march”^15,16^.

Skin, and more specifically its outermost layer, the epidermis, represents a first physical barrier preventing penetration of environmental factors such as pollutants, allergens, and microbes into the body. The epidermis is a stratified structure predominantly composed of keratinocytes^17^. Once committed to epidermal differentiation, undifferentiated basal keratinocytes migrate upwards and start producing structural proteins (primarily Keratins 1 and 10, involucrin, loricrin, and filaggrin) and lipids (mainly ceramides, glucosylceramides and cholesterol sulfate), necessary for the establishment of the *stratum corneum*^18^. Mostly composed of cross-linked, terminally differentiated cornified keratinocytes (the “cornified envelope” or CE) embedded in a mixture of intercellular lipids (ceramides, cholesterol, and free fatty acids), this uppermost layer of the epidermis is the cornerstone of skin barrier function^19^. Alteration in the formation of the cornified envelope caused by genetic mutations (e.g., filaggrin), lipid profiles, immune dysregulation, or environmental factors (e.g., protease-active allergens and pathogens) can result in skin barrier dysfunction and AD onset^20–23^.

Short-chain fatty acids (SCFA) are well-described metabolites derived from the fermentation of dietary fibre by colonic bacteria^24^. Dietary fibre and SCFA have been shown to modulate immune responses in a variety of inflammatory settings both locally in the gut and distally in organs such as the lung^25–27^ and skin^28,29^. SCFA can elicit their immunomodulatory capacity by acting as histone deacetylase (HDAC) inhibitors, binding to specific receptors (principally FFAR2 and FFAR3), or influencing cellular metabolism^25–27,30–35^. Their impact upon non-immune cells, on the other hand, is less well understood, with most research focusing on local effects in the gastrointestinal tract^36–40^. Of note, recent lines of evidence indicate that children and infants suffering from AD^41,42^, or prone to develop allergic sensitization later in childhood^43^, exhibit a gut microbiota characterized by a reduced capacity to produce SCFA, particularly butyrate. These findings support the hypothesis that a low fibre intake, characteristic of a westernized lifestyle, might underlie skin barrier dysfunction and a subsequent propensity for early allergen sensitization.

To test this hypothesis, we developed an experimental model whereby skin barrier impairment was triggered by exposing dorsal murine skin to ubiquitous house dust mite (HDM) allergens, which possess protease activity. Our model led to barrier disruption, percutaneous allergen sensitization, and ultimately onset of AD-like skin inflammation (ADLSI). We found that mice fed a high-fibre diet or administered SCFA orally (especially butyrate) had reduced allergen-induced skin barrier breach, less allergen ingress and sensitization, and hence diminished ADLSI. This protective effect was due to accelerated epidermal keratinocyte differentiation, characterized by enhanced production of key structural proteins and lipids, ultimately leading to strengthening of the *stratum corneum* following repeated allergen exposure. Butyrate promoted skin barrier function by directly shaping keratinocyte metabolism and eliciting a mitochondria-dependent differentiation program. Together, our results demonstrate that dietary fibre consumption and SCFA are pivotal factors that can help maintain skin barrier integrity and limit allergen sensitization, a finding of significant importance in the context of the atopic march.

## Results

### Dietary fibre and SCFA protect against allergen-induced ADLSI and systemic allergen sensitization

**Fig. 1:**
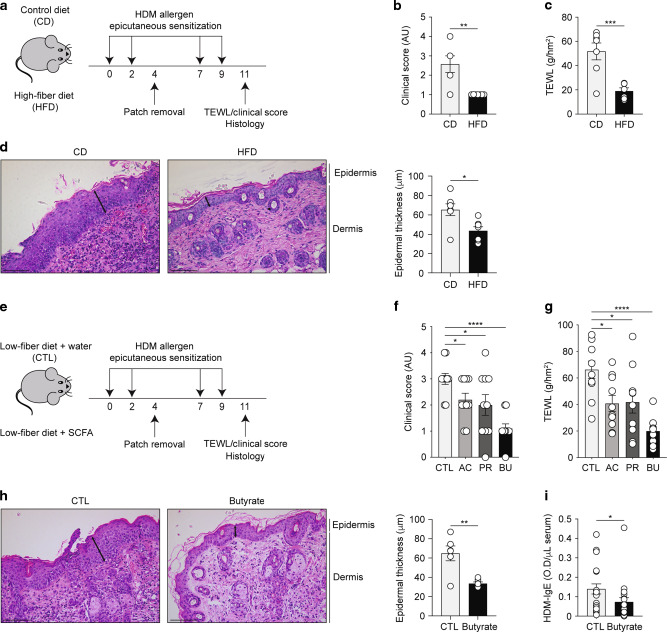
Dietary fibre and SCFA protect against allergen-induced ADLSI and systemic allergen sensitization. **a** Experimental model of skin barrier dysfunction and atopic dermatitis-like skin inflammation (ADLSI) using epicutaneous house dust mite (HDM) allergen sensitization (four times 15 μg HDM over 2 weeks) in control (cellulose; CD) or high-fibre diet (inulin; HFD) fed mice. **b** Disease severity assessment and **c** measurement of dorsal trans-epidermal water loss (TEWL) in CD or HFD-fed mice after 2 weeks of allergen sensitization. AU, arbitrary units. **d** Representative hematoxylin and eosin (H&E)-stained skin tissue from CD or HFD-fed mice after allergen exposure and quantification of epidermal thickness (distance from basal to upper granular layer, randomly measured 3 times per picture, 7–8 pictures per sample). Arrows represent representative sites for thickness measurement. Scale bars, 100 μm. **e** Experimental model of epicutaneous allergen sensitization in water control (CTL) or SCFA-treated mice. **f** Assessment of disease severity and **g** measurement of TEWL in CTL or SCFA-supplemented mice after allergen exposure. AC: Acetate, PR: Propionate, BU: Butyrate. **h** Representative H&E-stained skin tissue from CTL or butyrate-treated mice after allergen sensitization and quantification of epidermal thickness. Scale bars, 100 μm. **i** Levels of circulating HDM-specific IgE antibodies in serum at the end of the 2 weeks HDM epicutaneous sensitization model, as determined by ELISA. OD, optical density. Results are representative of data generated in three independent experiments in **b**–**d**, from at least three independent experiments in **h**, pooled from two (**f**, **g**) or four (**i**) independent experiments. All results are expressed as mean ± SEM (*n* = 7 per group in **b**–**d**; *n* = 10 per group in **f**, **g;** *n* = 6 per group in **h**; and *n* = 22 CTL and *n* = 20 butyrate in **i**). Statistical significance was determined with Student’s *t*-test (unpaired, two-tailed) in **b**–**d** and **f**–**h**, or Mann–Whitney test in **i**. *P = 0.05, **P = 0.01, ***P = 0.001, and ****P = 0.0001. See also Fig. S1.

Weanling mice were fed a low-fibre diet (less than 0.3% crude fibre) supplemented with either poorly-fermentable cellulose (control diet; CD) or inulin, a readily fermentable fibre with butyrogenic properties^26^ (high-fibre diet; HFD). To investigate the influence of HFD on skin barrier integrity and subsequent susceptibility to develop allergic disease, mice were repeatedly exposed to house dust mite (HDM) allergens on dorsal skin via an occlusive patch resulting in barrier disruption, allergen ingress and sensitization, and ultimately onset of ADLSI (Fig. 1a). Mice fed HFD exhibited less disease severity as compared to those on control diet (Figs. 1b and S1A) and improved barrier integrity as determined by measurement of trans-epidermal water loss (TEWL) (Fig. 1c). In comparison to skin from CD-fed mice, animals on HFD exhibited both decreased epidermal thickening and immune cell infiltration, as shown by analysis of hematoxylin and eosin (H&E)-stained lesional skin sections (Fig. 1d). Of note, previous data from our laboratory using this model demonstrated that without addition of HDM allergens, occlusion with a saline control patch does not lead to overt skin inflammation, or skin barrier dysfunction^44^. To address whether SCFA could replicate the beneficial effects of HFD, mice on low-fibre diet were given water (CTL) or SCFA orally (Fig. 1e). In line with the findings from Song et al. and Ta et al. reporting reduced SCFA levels in AD patients^41,42^, SCFA supplementation to low-fiber-diet fed mice alleviated ADLSI in a manner similar to that conferred by HFD, especially butyrate (Fig. 1f, g). Specifically, disease severity (Fig. 1f and S1B), TEWL (Fig. 1g), immune cell infiltration, and epidermal thickness (Fig. 1h) were all markedly improved in butyrate-treated animals. Quantification of circulating HDM-specific IgE revealed that butyrate supplementation mitigated systemic allergen sensitization (Fig. 1i). Of note, using ^13^C-labelled butyrate we could demonstrate that orally-delivered butyrate reaches the highly vascularized skin compartment within 45 min (Fig. S1C).

### Butyrate alters the skin transcriptome following allergen exposure

**Fig. 2:**
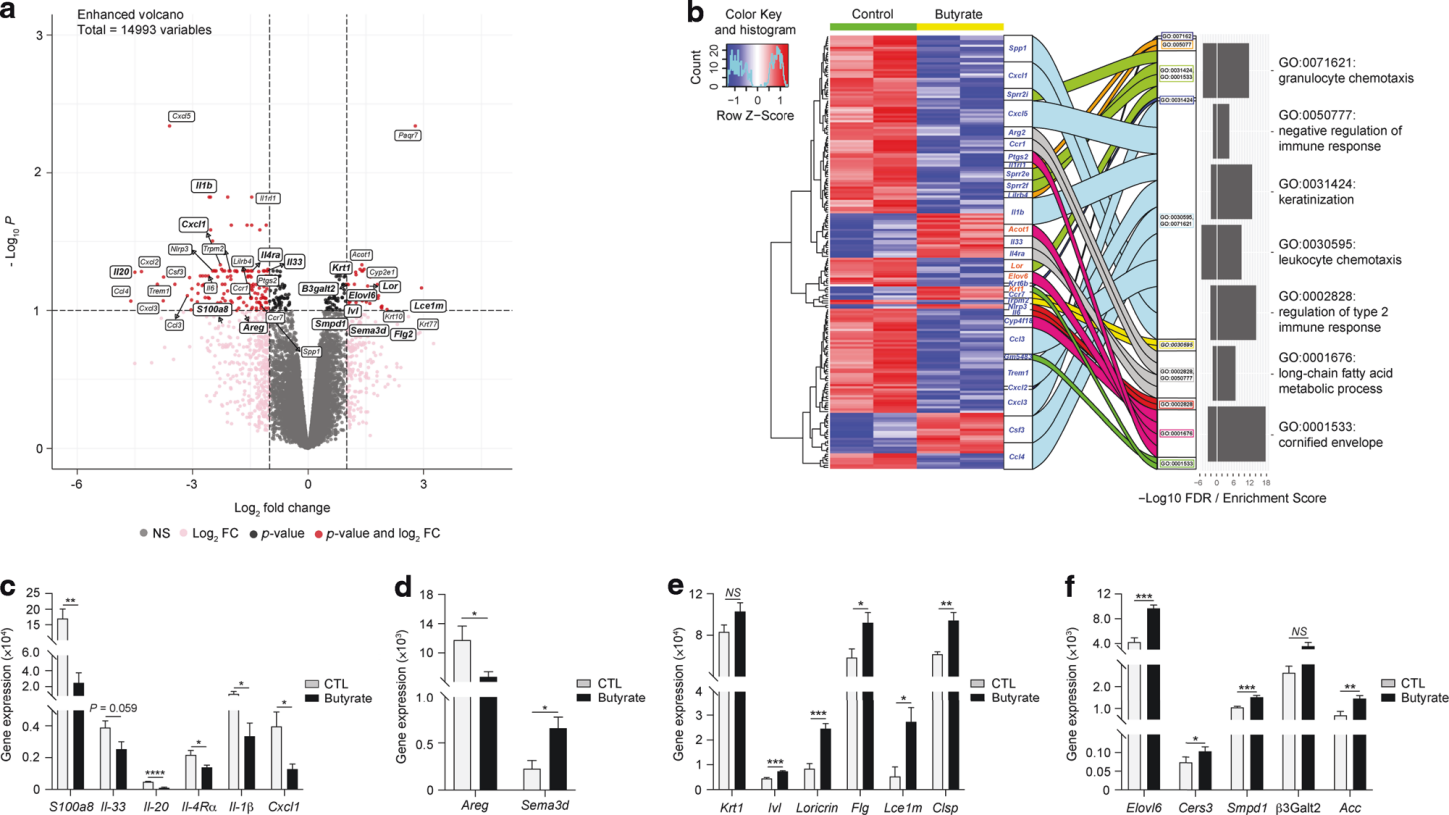
Butyrate alters the skin transcriptome following allergen exposure. **a** Volcano plot of differentially expressed genes (DEGs) after transcriptomics analysis of HDM allergen-sensitized skin from control or butyrate-treated mice (false detection rate (FDR) < 0.1; fold-change = 2). **b** Gene ontology (GO) analysis of lesional skin transcriptomics. Heatmap and alluvial plot of select DEGs (blue = decreased and red = increased in butyrate-treated mice) linked to GO IDs. Mirrored box plot indicates −log10 FDR on left and enrichment score on right for each GO ID. Gene expression analysis by quantitative RT-PCR of key genes involved in atopic dermatitis inflammation (**c**), itching pathway (**d**), keratinocyte differentiation and cornification (**e**), and lipid/ceramide synthesis and cornified lipid envelope formation (**f**) in skin from control (CTL) or butyrate-treated animals after 2 weeks HDM sensitization. Gene expression was normalized to *β-actin*. Results are representative of data from one experiment in **a** and **b**, and from three independent experiments in **c**–**f**. All results are expressed as mean ± SEM (*n* = 2 per group in **a**, **b**; *n* = 6 per group in **c**–**f**). Statistical significance was determined with Student’s *t*-test (unpaired, two-tailed) or Mann–Whitney test in **c**–**f**. *NS* = non-significant, *P = 0.05, **P = 0.01, ***P = 0.001, and ****P = 0.0001.

To gain mechanistic insights into the protective effects of butyrate, we performed an exploratory transcriptomics analysis of lesional skin from CTL and butyrate-treated mice. Differential gene expression analysis using Voom/Limma method showed a total of 195 genes that were differentially expressed (false detection rate (FDR) < 0.1, fold-change (FC) > 2) in skin from butyrate-treated mice compared to that of controls (Fig. 2a). Subsequent gene ontology (GO) enrichment analysis revealed pathways related to immunity and barrier function that were enriched in the skin of butyrate-treated mice. Immune-related GO terms included “negative regulation of immune response” (GO:0050777), “regulation of type 2 immune response” (GO:0002828), “leukocyte chemotaxis (GO:0030595), and “granulocyte chemotaxis” (GO:0071621) (Fig. 2b). The second cluster of pathways was linked to skin barrier function, as represented by GO terms “keratinization” (GO:0031424) and “cornified envelope” (GO:0001533) (Fig. 2b). To establish the relevance of these findings, we next interrogated how butyrate modulates immune-associated genes implicated in ADLSI onset and progression by performing quantitative reverse transcription-polymerase chain reaction (RT-PCR) on lesional skin biopsies from control and butyrate-treated mice. Consistent with the skin transcriptomics data, we found that immune genes implicated in human pediatric and adult AD pathogenesis were altered in our experimental model. The pro-T_H_2 mediators *Il-33* and *Il-4rα*, T_H_17/T_H_22-associated genes *S100a8* and *Cxcl1*, the epidermal hyperplasia-inducing *Il-20*, and the innate cytokine *Il-1β* were all reduced in the skin of butyrate-treated animals (Fig. 2c). Nerve elongation and retracting mediators produced by keratinocytes, and involved in itch control, were also altered by butyrate. Expression of amphiregulin (*Areg*), a nerve sprouting factor favoring itch, was significantly mitigated, whilst that of nerve repulsing factor semaphorin 3D (*Sema3d*) was enhanced in lesional skin of butyrate-treated mice (Fig. 2d).

Next, we investigated the influence of butyrate upon genes related to keratinocyte differentiation and skin barrier function, the second main pathway highlighted by our exploratory transcriptomics analysis. We found that lesional skin from butyrate-exposed mice expressed higher levels of genes characteristic of differentiating and terminally differentiated keratinocytes in addition to CE formation. Indeed, expression of involucrin (*Ivl*), loricrin (*Lor*), filaggrin (*Flg*), late cornified envelope 1 m (*Lce1m*), and calmodulin-like skin protein (*Clsp*)^45^ were all enhanced compared to control mice (Fig. 2e). CE not only relies on the aforementioned proteins for its functional integrity but also requires keratinocyte-derived lipids such as ceramides and free fatty acids (FFA) to provide a tight, hydrophobic barrier preventing allergen ingress and trans-epidermal water loss^46^. We therefore assessed the expression of genes for several key enzymes involved in FA biosynthesis (acetyl-CoA carboxylase (*Acc*)) and elongation into long-chain fatty acids (LCFA) (elongation of very long-chain fatty acids protein 6 (*Elovl6*)), as well as those critical for ceramide synthesis (ceramide synthesis 3 (*Cers3*), acid sphingomyelinase (*Smpd1*), and β-1,3-galactosyltransferase 2 (*β3Galt2*)). As shown in Fig. 2f, we found that skin from butyrate-treated mice expressed elevated levels of these genes, a finding consistent with the transcriptomics analysis indicating an enrichment in the GO term “Long-chain fatty acids metabolic process” (GO:0001676) (Fig. 2b). Altogether, these results indicate that butyrate administration alters the keratinocyte response to HDM and alleviates the associated downstream inflammatory cascade. Of note, similar results were observed in lesional skin from mice fed HFD (data not shown).

### Early cutaneous immune responses to HDM allergens are blunted in butyrate-treated animals

**Fig. 3:**
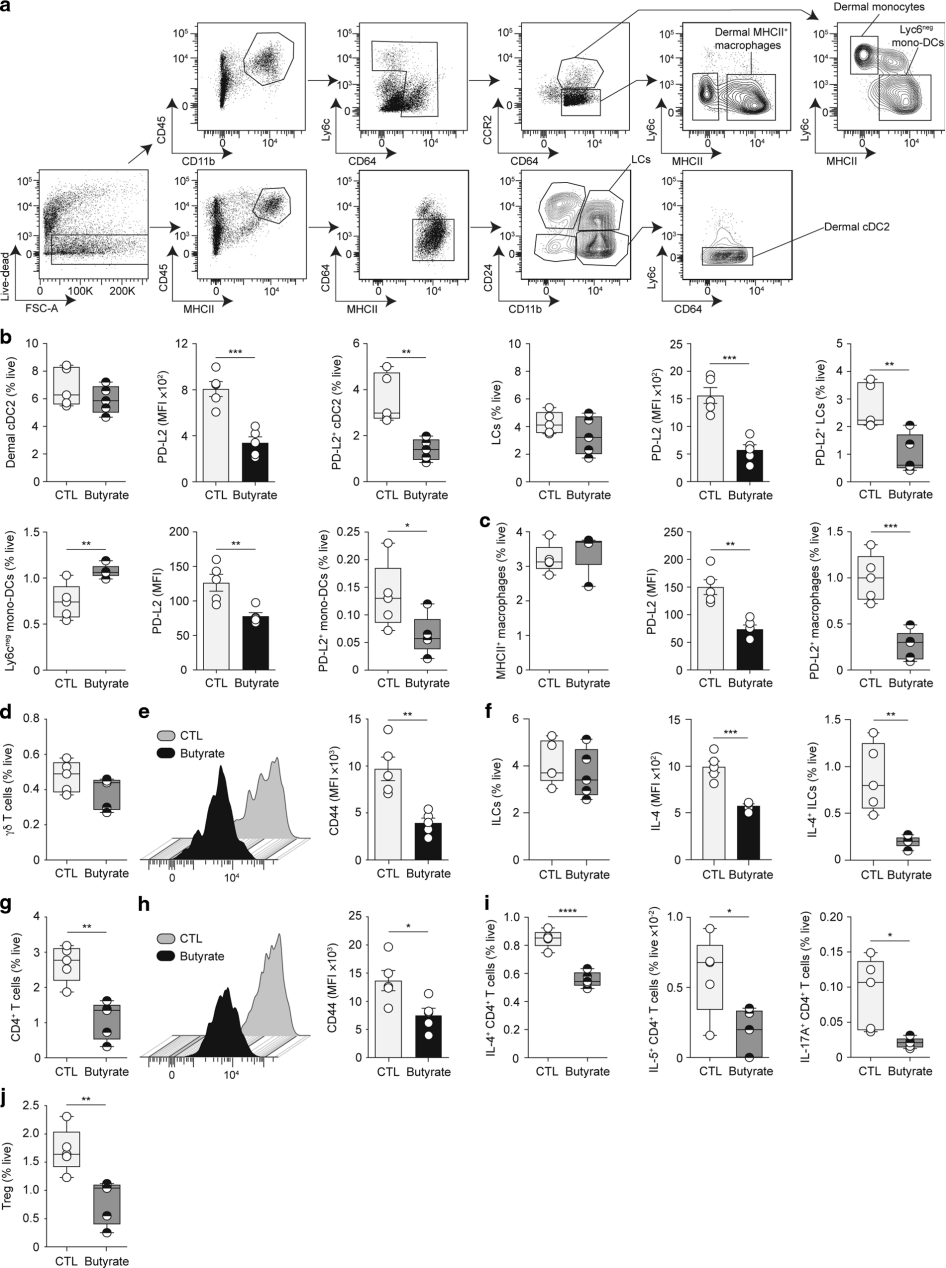
Early cutaneous immune responses to HDM allergens are blunted in butyrate-treated animals. **a** Gating strategy for flow cytometry analysis of the main antigen-presenting cells in the skin of control (CTL) or butyrate-supplemented mice. **b** Frequencies of main dendritic cell (DC) subsets (CD11b^+^ cDC2, Langherans cells (LCs), and Ly6c^neg^ mono-DCs) and their surface expression of Th2 activation marker program death-ligand 2 (PD-L2) in the skin of CTL and butyrate-treated animals. MFI, mean fluorescence intensity. **c** Frequencies and PD-L2 expression (MFI) by the most prominent skin macrophage population. **d**, **e** Frequencies of skin γδT cells and their surface expression of activation marker CD44 (MFI) in CTL and butyrate-treated mice. **f** Frequencies of total and IL-4-producing innate lymphoid cells (ILCs) in the skin, and their expression of IL-4 (MFI). Frequencies of CD4^+^ T lymphocytes in the skin (**g**) and their surface expression of CD44 (MFI) (**h**), as well as frequencies of IL-4-, IL-5, and IL-17A-producing CD4^+^ T cells (**i**). **j** Frequencies of Foxp3^+^ T regulatory cells (Tregs) in the skin. Data were determined by flow cytometry after two topical HDM allergen sensitizations. Results are representative of data from two to three independent experiments in **b**, **c**, from two independent experiments in **d**–**f** and **i**, and from at least three independent experiments in **g**, **h** and **j**. All results are expressed as mean ± SEM (*n* = 5 per group in **b**–**h** and **j**; *n* = 6 per group in **I**). Statistical significance was determined with Student’s *t*-test (unpaired, two-tailed) in **b**–**j**, or Mann–Whitney test in **b**. *P = 0.05, **P = 0.01, ***P = 0.001, and ****P = 0.0001. See also Fig. S2.

The exploratory transcriptomics data indicated that butyrate supplementation affects immune responses generated during ADLSI. We and others have shown that SCFA can influence immune cell functionality^25,26,30,47,48^. We thus examined how butyrate shaped early immune responses to HDM (1-week post-allergen exposure), before symptoms manifest, to assess the influence of butyrate upon ADLSI onset. Flow cytometric phenotyping of the skin revealed that butyrate did not alter the composition of cutaneous antigen-presenting cell (APC) populations after HDM exposure (see Fig. 3a for gating strategy). Dermal CD11b^+^ conventional dendritic cells (cDC) 2, Langerhans cells (LCs), Ly6c^neg^ monocyte-derived DCs (Fig. 3b), and major histocompatibility complex II (MHCII)^+^ dermal macrophages (Fig. 3c) were found in similar frequencies in allergen-exposed skin of CTL and butyrate-treated mice. We found, however, that skin APCs from butyrate-administered animals exhibited lower expression of programmed death-ligand 2 (PD-L2) (Fig. 3b, c), a surface marker shown to be important for driving cutaneous T_H_2 cell responses^49,50^. Moreover, frequencies of PD-L2-expressing APCs were also diminished (Fig. 3b, c), indicating an impairment in the ability of skin APCs from butyrate-exposed animals to promote T_H_2 differentiation. The proportions of γδ T cells, a population found in murine but not human skin (Fig. 3d), and innate lymphoid cells (ILCs) (Fig. 3f), two pivotal modulators of AD onset^51,52^ were unaltered by butyrate. Yet, as for the APC subsets, their activation state was dampened, as represented by lower expression of CD44 on γδ T cells (Fig. 3e) and a blunted capacity of ILC2 to produce IL-4 (Fig. 3f). IL-5 production was, however, unaltered (data not shown). T_H_17 inflammation has been linked to the onset of AD^53,54^. We thus assessed the production of IL-17A by ILCs and γδ T cells, two major IL-17-producing populations^52^. We discovered that (i) these cells secreted limited amounts of IL-17A in our experimental model of ADLSI; and (ii) no significant differences were observed between control and butyrate-treated animals (data not shown). In addition, we found that frequencies of CD4^+^ T lymphocytes were significantly diminished in HDM-exposed skin of mice treated with butyrate (Fig. 3g). CD4^+^ T cells were not only reduced in number but their activation state was decreased (Fig. 3h) and their ability to secrete IL-4, IL-5, and IL-17A in response to ex vivo stimulation was also impaired (Fig. 3i). These results demonstrate that butyrate-treated animals exhibit a blunted early cutaneous immune response following HDM exposure. The immunomodulatory properties of SCFA, particularly butyrate, have been linked to their ability to induce CD4^+^ T regulatory cells (Treg)^29^. We thus assessed whether butyrate treatment altered skin Treg in the model of ADLSI. We found that allergen-exposed skin from butyrate-administered animals harbored less Treg (Fig. 3j). These findings indicate that the mitigated ADLSI observed in butyrate-treated mice is not attributable to an elevated Treg response. Importantly, we found that butyrate did not change the skin immune cell environment before allergen exposure (Fig. S2A–F), indicating that butyrate-mediated alterations of cutaneous immune responses manifested only after allergen-driven skin injury occurred. The beneficial effects of butyrate on ADLSI could result from (i) the direct dampening of allergen-driven T_H_2 responses by this SCFA, a well-described phenomenon^29^; and/or (ii) an improved skin barrier function limiting allergen penetration and the downstream inflammatory consequences. To test these hypotheses and determine whether butyrate has direct immunomodulatory effects upon cutaneous immune responses to HDM allergens, we next performed similar experiments as those described in Fig. 3 however HDM allergens were instilled intradermally, where most skin APCs reside, and not topically. By using such an approach, we bypassed the epidermal barrier and delivered the allergens directly to local APCs. This strategy eliminates the potential role of butyrate upon skin barrier function, thereby allowing a direct assessment of the influence of butyrate upon skin immune responses to HDM allergens. When delivered intradermally and not topically, HDM allergen-driven cutaneous T_H_2 responses were no longer impaired in the skin of butyrate-treated animals (Fig. S2G–M). This finding indicates that the improved ADLSI observed in butyrate-exposed mice is likely not resulting from a direct effect on immune cells.

### Butyrate promotes skin barrier function

**Fig. 4:**
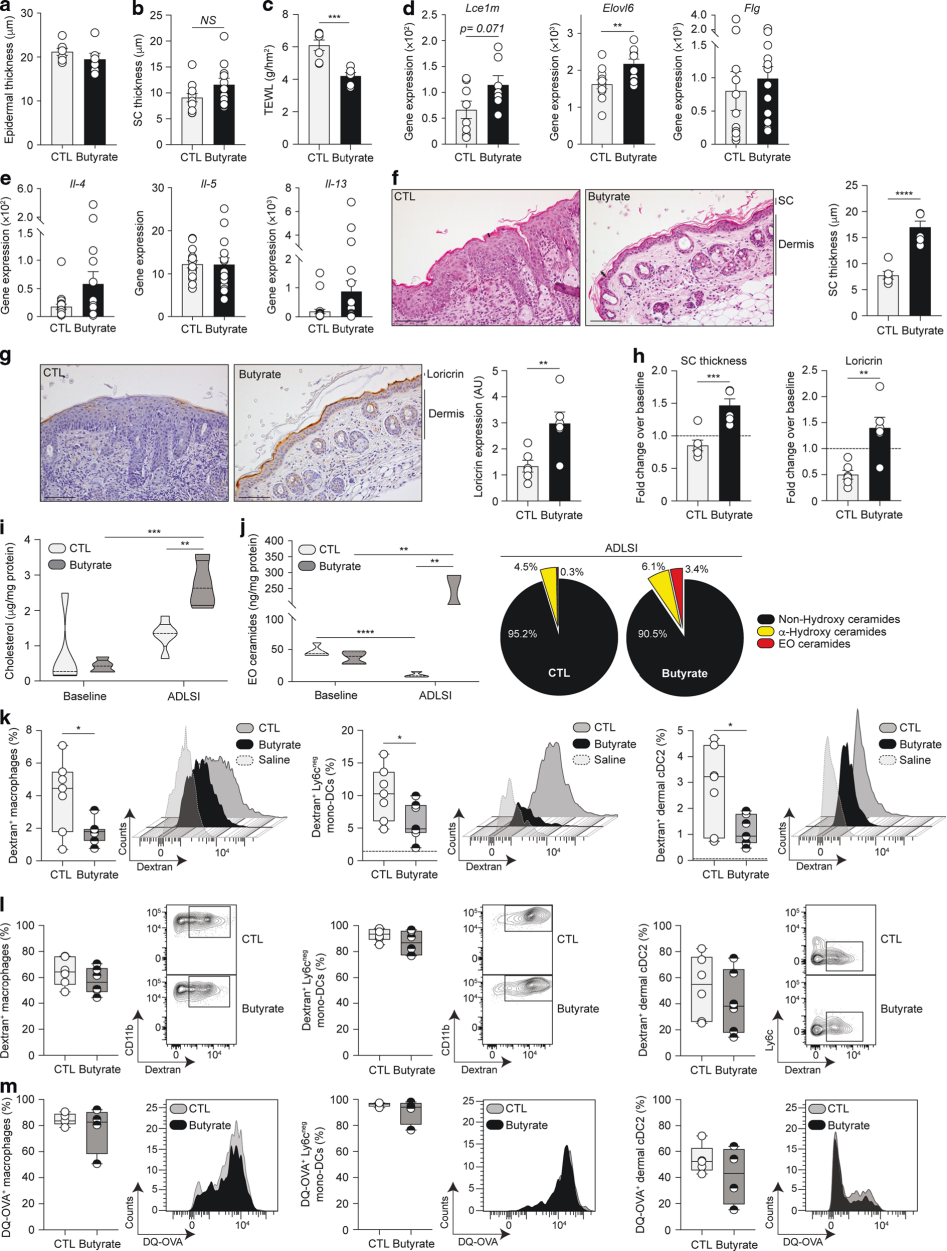
Butyrate promotes skin barrier function. **a** Quantification of baseline epidermal thickness in water control (CTL) or butyrate-treated mice before allergen sensitization. **b** Quantification of *stratum corneum* (SC) thickness following analysis of representative hematoxylin and eosin (H&E)-stained skin tissue before HDM allergen exposure. **c** Measurement of baseline dorsal transepidermal water loss (TEWL) in control or butyrate-treated mice before allergen exposure. **d** Expression of key components of the cornified envelope by fluorescence-activated cell sorting (FACS)-purified epidermal CD326^+^ keratinocytes isolated before allergen sensitization from CTL mice or butyrate-treated mice, as determined by quantitative RT-PCR. **e** Expression of T_H_2 cytokines in the skin at baseline, as determined by quantitative RT-PCR. Gene expression was normalized to *β-actin* in **d** and **e**. **f** Representative H&E-stained skin tissue from CTL or butyrate-treated mice after 2 weeks of HDM allergen exposure and corresponding quantification of *stratum corneum* (SC) thickness (randomly measured 3 times per picture, 7–8 pictures per sample). Arrows represent representative sites for SC thickness measurement. Scale bars, 100 μm. **g** Representative immunohistochemistry-stained skin tissue from CTL or butyrate-supplemented mice after 2 weeks of HDM allergen sensitization and corresponding quantification of loricrin expression (represented by a light-to-dark brown staining). Loricrin staining was measured in the entire picture, with 4 pictures per sample. Scale bars, 100 μm. **h** Fold-change (2 weeks HDM-sensitized over baseline) SC thickness and loricrin expression in the skin of CTL or butyrate-exposed animals. Dotted line represents an arbitrary value for baseline fold-change. **i** Absolute quantities of cholesterol in the skin of naive (“baseline”) and HDM allergen-sensitized (“ADLSI”) CTL and butyrate-treated mice, as determined by ultrahigh performance supercritical fluid chromatography coupled to quadrupole time-of-flight mass spectrometry with electrospray ionisation (UHPSFC/ESI-QTOF-MS^E^). **j** Distribution of the main classes of ceramides and absolute quantities of Ester-linked-Omega-hydroxy (EO) ceramides, as determined by ultrahigh performance liquid chromatography coupled to tandem mass spectrometry with electrospray ionisation (UPLC/ESI-MS/MS). **k** Frequencies and representative histograms of FITC-dextran-positive MHCII^+^ dermal macrophages, Ly6c^neg^ mono-DC, and CD11b^+^ cDC2 in the skin of two-time HDM allergen-sensitized control (CTL) or butyrate-treated animals after one topical administration of 100 μg FITC-dextran (10 KDa). Saline = HDM-exposed skin without FITC dextran administration (saline-treated). Frequencies and representative histograms of FITC positive cells in the skin of two-time HDM allergen-sensitized CTL or butyrate-supplemented mice after one intradermal administration of 100 μg FITC-dextran (**l**) or 100 μg DQ-OVA (**m**). Results are representative of data from 3 independent experiments in **a** and **C**, pooled from 2 independent experiments in **b**, **d**, pooled from 4 independent experiments in **e**, from at least three independent experiments in **f**–**h**, from one independent experiment in **i**, **j**, and from two independent experiments in **k**–**m**. All results are expressed as mean ± SEM (*n* = 7 CTL and *n* = 6 butyrate in **a** and **c**; *n* = 11 per group in **b**; *n* = 8/12 for CTL and *n* = 7/11 for butyrate in **d**; *n* = 16/23 for CTL and *n* = 15/23 for butyrate in **e**; *n* = 6 per group in **f**–**h** and **l**; *n* = 5 CTL “baseline”, 5 CTL “ADLSI”, 5 butyrate “baseline”, and 4 butyrate “ADLSI” in **i**; *n* = 5 CTL “baseline”, 4 CTL “ADLSI”, 5 butyrate” baseline”, and 3 butyrate “ADLSI” in **j**; *n* = 7 per group in **k**; *n* = 5 CTL and 4 butyrate in **m**). Statistical significance was determined with Student’s *t*-test (unpaired, two-tailed) in **a**–**m**, or Mann–Whitney test in **e** and **k**. *P = 0.05, **P = 0.01, ***P = 0.001, and ****P = 0.0001.

To test whether the protective effects of butyrate observed in the ADLSI model were the consequence of an improved skin barrier (thereby restricting allergen ingress), we first assessed skin morphology and barrier function before allergen exposure, aware that cutaneous immune responses were unaltered in these conditions (Fig. S2a–f). We found that even though butyrate did not lead to observable alterations in epidermal thickness at baseline (Fig. 4a), naive skin of butyrate-exposed mice had the tendency to exhibit a thicker *stratum corneum* (SC), the outermost protective layer of the epidermis, potentially indicative of enhanced barrier function (Fig. 4b). To functionally test this, we measured TEWL and found that mice exposed to butyrate had better skin barrier integrity at baseline, prior to allergen exposure, than control mice (Fig. 4c). In support of this, epidermal keratinocytes isolated from healthy skin of butyrate-exposed mice showed elevated expression of several key barrier function genes before allergen encounter (Fig. 4d). T_H_2 cytokines have been shown to exert inhibitory effects on skin barrier function and epidermal differentiation complex genes^23,55^. Importantly, T_H_2 cytokine expression levels in the naive skin remained similar between control and butyrate-treated animals, ruling out a role for T_H_2 cytokines in the butyrate-driven improved skin barrier function observed at baseline (Fig. 4e). Overall, these data indicate that although no differences were observed in the immune environment (Figs. 4e and S2), skin from butyrate-treated mice showed signs of enhanced barrier function prior to allergen exposure.

We next assessed the skin after HDM exposure to determine how these aforementioned alterations in skin morphology and barrier function evolved. Skin from butyrate-treated mice exhibited a significantly thicker SC after allergen exposure, a difference compared to control skin that was more pronounced than at baseline (Fig. 4f). Loricrin represents nearly 80% of the SC protein content and is pivotal for reinforcing skin barrier function due to its cross-linking and binding properties. The skin of HDM-exposed control mice had diminished and disrupted loricrin expression compared to butyrate-exposed animals whose skin exhibited a continuous layer (Fig. 4g). Loricrin expression was unaltered in naive healthy skin (data not shown). T_H_2 cytokines produced after allergen sensitization are known to weaken skin barrier function by decreasing the production of various constituents of the SC (e.g., loricrin, filaggrin, and lipids)^23,55,56^, an event we observed in control animals when comparing SC thickness and loricrin expression levels before and after allergen exposure, as represented by a fold-change over baseline below one (Fig. 4h). Contrastingly, in butyrate-treated mice, SC thickness and loricrin expression both significantly increased after HDM exposure compared to baseline healthy skin (Fig. 4h). Lastly, we investigated whether lipids, the other key constituent of SC, were altered by butyrate. Lipidomics analysis of naive and lesional skin revealed that skin from butyrate-exposed mice contained greater amounts of cholesterol (Fig. 4i) and ceramides, particularly ester-linked omega-hydroxy (EO) ceramides (Fig. 4j). EO ceramides are pivotal to skin barrier function, and their deficiency is linked with AD^57,58^. EO ceramide quantities decreased in the skin of control mice after allergen challenge, a phenomenon also observed in AD patients^59–61^, while they increased in butyrate-treated animals, indicative of improved barrier function following allergen exposure (Fig. 4j).

To determine if these morphological changes had any functional relevance, we examined whether percutaneous antigen (Ag) penetration was altered in mice treated with butyrate. To achieve this, we employed two experimental approaches and used 10 KDa fluorescein isothiocyanate (FITC)-dextran, a fluorescently-labelled Ag whose size is similar to major HDM allergens. First, we applied FITC-dextran onto the skin (combined with HDM to mimic the initial allergen encounter) to assess Ag penetration and uptake by local APCs. We found that frequencies of FITC-positive dermal MHCII^+^ macrophages, Ly6c^neg^ monocyte-derived DCs, and dermal CD11b^+^ cDC2 were all significantly reduced in the skin of butyrate-treated mice, indicative of a blunted allergen ingress (Fig. 4k). To address whether these findings were the consequence of an enhanced epidermal barrier function or a defect in APC functionality, we performed a second set of experiments. FITC-dextran was delivered intradermally, thereby bypassing the epidermis and its barrier function, allowing for a direct assessment of APC functionality. In this setting, Ag-positive cells were no longer reduced in butyrate-treated mice indicating normal Ag uptake capacity (Fig. 4l). To further examine Ag processing, we used DQ-ovalbumin (OVA), a molecule that becomes fluorescent only when proteolytically degraded. Dermal APCs from butyrate-administered mice exhibited similar Ag processing capacities compared to control APCs (Fig. 4m). These findings show that butyrate limits percutaneous Ag penetration by strengthening epidermal barrier integrity, without altering skin APCs functionality (or their proportions, as shown in Fig. 3). Altogether, these results indicate that butyrate enhances skin barrier function at baseline but also strengthens it following repeated allergen exposure, two key events that restrict allergen ingress and sensitization, and thus mitigate onset and development of ADLSI.

### Butyrate promotes terminal differentiation of epidermal keratinocytes

**Fig. 5:**
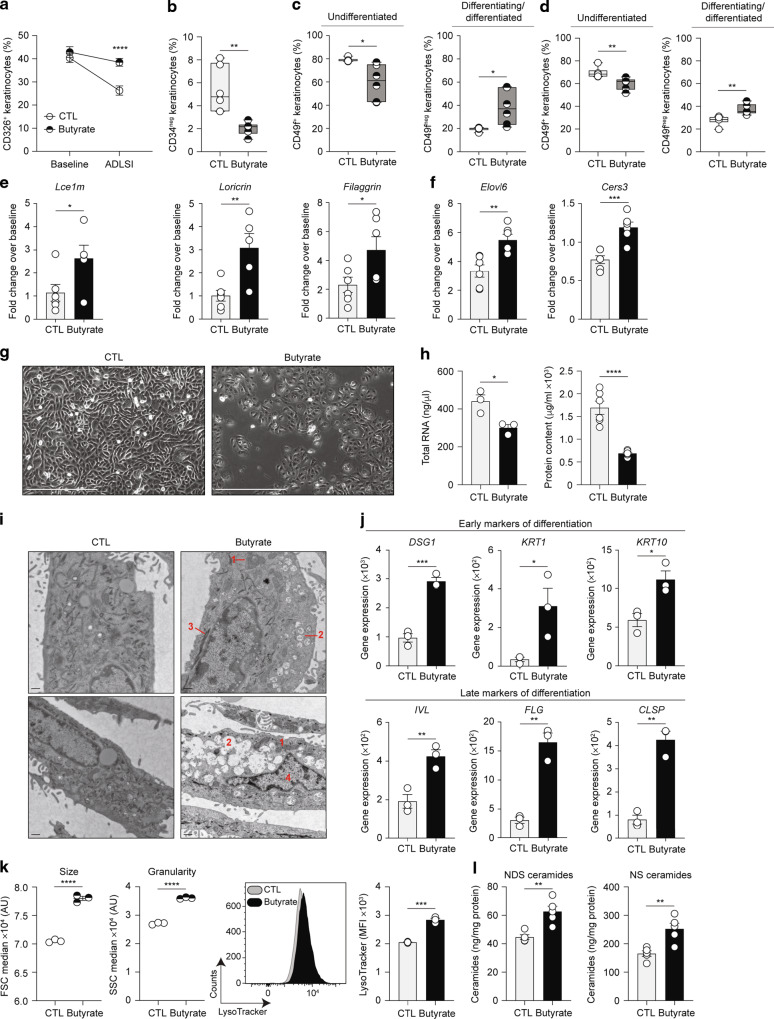
Butyrate promotes terminal differentiation of epidermal keratinocytes. **a** CD326^+^ keratinocytes in the skin of naive (“baseline”) and two-time house dust mite (HDM) allergen-sensitized (“ADLSI”) CTL and butyrate-exposed mice, as assessed by flow cytometry. **b** CD34^+^ hair bulge keratinocytes in HDM allergen-sensitized skin from water control (CTL) or butyrate-supplemented mice, as assessed by flow cytometry. CD49f^+^ basal CD326^+^ keratinocytes and CD49f^neg^ differentiating/differentiated CD326^+^ keratinocytes in baseline (**c**) or two-time HDM allergen-sensitized (**d**) skin from CTL or butyrate-treated mice, as determined by flow cytometry. Fold-change expression over baseline of key components of the cornified envelope (**e**) or key enzymes for generation of long-chain fatty acids and ceramides (**f**) in fluorescence-activated cell sorting (FACS)-purified CD326^+^ CD34^neg^ epidermal interfollicular keratinocytes isolated from two-time HDM allergen-sensitized control (CTL) or butyrate-treated mice, as determined by quantitative RT-PCR. Gene expression was normalized to *β-actin*. **g** Representative phase-contrast micrographs of primary human epidermal keratinocytes (HEK) either vehicle (CTL) or butyrate (500 μM)-supplemented for 48 h. Scale bars, 400 μm. **h** Total RNA and protein contents from HEK cultures. **i** Representative transmission electron microscopy micrographs of HEK cultures. Annotations: 1 = enlarged mitochondria; 2 = lysosomes; 3 = tonofibrils; 4 = degenerating nucleus. Scale bars, 500 nm. **j** Expression of skin barrier genes in HEK cultures, as determined by quantitative RT-PCR. Gene expression was normalized to *β-ACTIN*. **k** Median size (FSC, Forward scatter) and granularity (SSC, Side scatter) of HEK cultures, and assessment of the presence of acidic lysosome-like organelles by flow cytometry using LysoTracker probe. MFI, mean fluorescence intensity. **l** Quantification of ceramide production by HEK cultures, as determined by UPLC/ESI-MS/MS. Results are representative of data pooled from three (group “baseline”) and four (group “ADLSI”) independent experiments in **a**, from three independent experiments in **b** and **d**, from two independent experiments in **c**, **e**, and **f**, from at least three independent experiments in **g**, **h**, **j**, and **k**, and from one experiment in **i** and **l**. All results are expressed as mean ± SEM (*n* = 18 per group in “baseline” and *n* = 24 CTL and *n* = 23 Butyrate in “ADLSI” in **a**; *n* = 6 per group in **b**–**e**, and **f**; *n* = 3–6 per group in **g**, **h**, **j**, and **k**; *n* = 2 per group in **i**; *n* = 5 per group in **l**). Statistical significance was determined with Student’s *t*-test (unpaired, two-tailed) in **a**–**f**, **h**, and **j**–**l**. *P = 0.05, **P = 0.01, ***P = 0.001, and ****P = 0.0001. See also Fig. S3.

The increase of SC thickness, loricrin expression, and ceramide abundance observed in the skin of butyrate-treated mice, particularly following allergen insult (Fig. 4h, j), point towards an effect of butyrate upon epidermal keratinocytes. Quantification of CD34^neg^ CD326^+^ interfollicular keratinocytes revealed that baseline skin from mice treated with butyrate contained similar proportions of epidermal keratinocytes (Fig. 5a), a finding consistent with the similar epidermal thickness shown in Fig. 4a. Of note, keratinocyte frequencies diminished in both groups in lesional ADLSI skin, yet by significantly less in animals exposed to butyrate. This finding is likely due to the fact that these mice exhibit a reduced recruitment of inflammatory cells in the skin, hence the elevated proportion of non-immune cells such as keratinocytes (Fig. 5a). Accordingly, butyrate-supplemented animals had less CD34^+^ hair bulge keratinocytes after allergen exposure (Fig. 5b), a population that has been shown to expand after skin injury^62^. Based on their expression of CD49f, epidermal keratinocytes can be classified into undifferentiated (CD49f^+^) or differentiating/differentiated (CD49f^neg^) keratinocytes^62^. We found that butyrate supplementation led to elevated proportions of CD49f^neg^ differentiated keratinocytes in naive skin (Fig. 5c), confirming our previous data showing an enhancement of baseline skin barrier function by butyrate (Fig. 4c). This difference in keratinocyte differentiation state found in the healthy skin of butyrate-treated mice was maintained after allergen exposure (Fig. 5d). To functionally assess the differentiation status of epidermal keratinocytes, we next isolated these cells from murine skin and looked at expression levels of key genes involved in skin barrier function. We found that, already at baseline (Fig. 4d), but especially after allergen exposure, keratinocytes from butyrate-treated mice exhibited a significantly augmented expression of *Lce1m*, *Loricrin*, and *Filaggrin* (Fig. 5e), as well as enzymes involved in LCFA and ceramide synthesis (Fig. 5f) compared to controls. These findings demonstrate that butyrate alters the fate of epidermal keratinocytes in vivo, promoting their differentiation and the strengthening of the cornified envelope at baseline, and even more so after allergen exposure.

To elucidate whether butyrate drives these effects by acting directly on keratinocytes and to assess the relevance of our findings to human cells, we next treated primary human epidermal keratinocytes (HEK) in vitro with butyrate and evaluated ensuing morphological and functional changes. Phase-contrast micrographs of HEK cultures indicated butyrate supplementation induced morphological alterations characteristic of keratinocyte differentiation (Fig. 5g). Specifically, butyrate-treated HEK were less confluent, larger, exhibited a reduced nucleus to cytoplasm ratio, intercellular spaces were less apparent, and cells lost their hexagonal, cobble-stone shape appearance distinctive of undifferentiated cells^63,64^. We next quantified total RNA and protein content from control or butyrate-treated HEK cultures as calcium-mediated HEK differentiation was shown to inhibit both RNA and protein synthesis^63^. In line with the observed morphological changes, we found that butyrate-supplemented HEK produced less RNA and protein compared to control HEK (Fig. 5h). Transmission electron microscopy (TEM) micrographs confirmed our findings and showed that butyrate-treated HEK exhibit more frequent tonofibrils, numerous lysosomes or lysosome-like organelles, enlarged mitochondria, and degenerating nucleus, all characteristic of differentiating HEK (Fig. 5i). Gene expression analysis corroborated these microscopy findings. HEK treated with butyrate expressed higher levels of both, “early” markers of differentiation such as Desmoglein-1 (*DSG1)*, Keratin-1 (*KRT1), KRT10*, as well as “late” markers of differentiation such as *IVL*, *FLG*, and *CLSP* (Fig. 5j). Butyrate also potentiated calcium-induced differentiation (Fig. S3A). This finding is of particular significance in vivo considering that the epidermis exhibits a calcium gradient with low calcium concentrations in the basal layer, where keratinocytes are undifferentiated, and high concentrations in the more superficial layers of the epidermis where keratinocytes undergo differentiation. Flow cytometry analysis further confirmed the enhanced differentiated state of butyrate-treated HEK, which exhibited larger size, granularity, and greater contents of acidic organelles (such as the lysosomes observed by TEM) as quantified using LysoTracker dye (Fig. 5k). Lipidomics analysis of HEK cultures revealed that HEK supplemented with butyrate produced more NS and NDS ceramides (Fig. 5l). These two ceramide classes are produced by keratinocytes in culture and can be considered an in vitro proxy for EO ceramides which cannot be found in cells grown on monolayer as their production depends on enzymes only expressed in a stratified epidermis. The abundance of EO ceramides is critical for barrier integrity in vivo and this increased NS and NDS production in vitro aligns with the elevated EO ceramide levels detected in the skin of butyrate-exposed mice (Fig. 4j).

### Butyrate reprograms keratinocyte metabolism to induce mitochondria-dependent epidermal differentiation

**Fig. 6:**
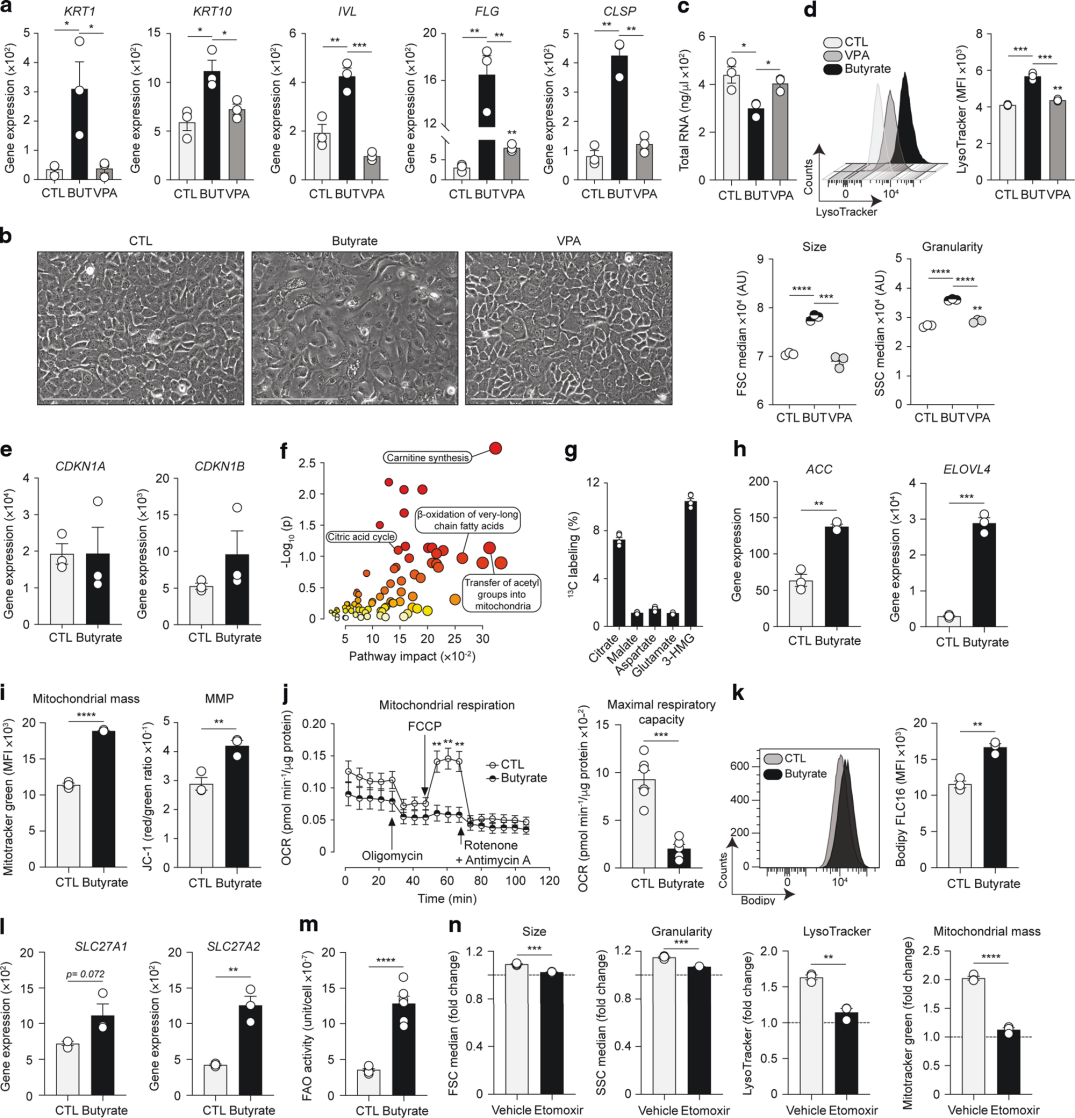
Butyrate reprograms keratinocyte metabolism to induce mitochondria-dependent epidermal differentiation. **a** Expression of skin barrier genes in HEK cultures either vehicle (CTL), 500 μM butyrate (BUT), or 500 μM valproate (VPA)-treated for 48 h, as determined by quantitative RT-PCR. **b** Representative phase-contrast micrographs of HEK cultures. Scale bars, 200 μm. **c** Total RNA quantification from HEK cultures. **d** Median size, granularity, and LysoTracker expression in HEK cultures, as determined by flow cytometry. MFI, mean fluorescence intensity. **e** Gene expression of *CDKN1A* and *CDKN1B* in HEK cultures, as determined by quantitative RT-PCR. **f** Pathway enrichment (based on the small molecule pathway database (SMPD)) and topology analyses of the multiple pathway targeted metabolomics data on FACS-isolated murine CD326^+^ keratinocytes highlighting several robustly altered metabolic pathways, as determined using MetaboAnalyst 5.0 software. **g** ^13^C enrichment in several intermediates and products of the citric acid cycle in HEK supplemented with ^13^C-butyrate for 24 h, as determined by Hydrophilic Interaction Liquid Chromatography coupled to high-resolution mass spectrometry. **h** Expression of malonyl-CoA-generating enzyme *ACC* and fatty acid elongation enzyme *ELOVL4* in HEK cultures, as determined by quantitative RT-PCR. **i** Determination of mitochondrial mass and membrane potential (MMP) of HEK cultures, as assessed by flow cytometry using MitoTracker green and bivariate dye JC-1, respectively. **j** Mitochondrial respiration of CTL or butyrate-treated HEK for 48 h, as determined by oxygen consumption rate (OCR) Seahorse assays. FCCP, trifluoromethoxy carbonylcyanide phenylhydrazone. **k** Measurement of long-chain fatty acids (LCFA) uptake by HEK, as determined by flow cytometry using a synthetic fluorescent LCFA (Bodipy FLC16). **l** Expression of LCFA transporters and activators *SLC27A1* and *SLC27A2* in HEK cultures, as assessed by quantitative RT-PCR. **m** Quantification of fatty acid oxidation (FAO) activities in HEK cultures. **n** Effect of low-dose etomoxir (20 μM) on median size, granularity, lysosome contents, and mitochondrial mass in cultures, as determined by flow cytometry. Results are expressed as fold-change over CTL in the absence (“vehicle”) or presence of etomoxir. For data in **a**, **e**, **h**, and **l**, gene expression was normalized to *β-ACTIN*. Results are representative of data from two to three independent experiments in **a**, at least three independent experiments in **b**, **d**, **h**, **k**, and **l**, three independent experiments in **c**, **i**, **j**, **m**, and **n**, and from one experiment in **f**–**g**. All results are expressed as mean ± SEM (*n* = 3 per group in **a**–**e**, **k, l**, and **n**; *n* = 5 per group in **f**–**g**; *n* = 6 per group in **m**; *n* = 6 for CTL and *n* = 5 for butyrate in **j**). Statistical significance was determined with Student’s *t*-test (unpaired, two-tailed). *P = 0.05, **P = 0.01, ***P = 0.001, and ****P = 0.0001. See also Fig. S4.

We next set out to elucidate the mechanisms underlying the pro-differentiating effects of butyrate upon epidermal keratinocytes. SCFA can exert their effects via three main pathways: (i) binding of specific receptors; (ii) acting as HDAC inhibitors; and (iii) altering cellular metabolism. Keratinocytes have been shown to express very low levels of *FFAR2*, *FFAR3*, and *GPR109a*, the main receptors for SCFA, while butyrate has been demonstrated to influence gene expression in keratinocytes by regulating histone acetylation^65^. We thus examined whether HDAC inhibition (HDACi) by SCFA governed the enhanced keratinocyte differentiation observed in our model. We first found that valproate (VPA), another SCFA which like butyrate strongly inhibits class I and IIa HDACs, did not influence expression of genes involved in keratinocyte differentiation (Fig. 6a). Phase-contrast microscopy and flow cytometry analyses confirmed that VPA had no detectible effects upon HEK morphology (Fig. 6b), RNA synthesis (Fig. 6c), size, granularity, and lysosomal contents (Fig. 6d) compared to butyrate. These data suggest that butyrate does not modulate HEK differentiation via an HDACi mechanism. We further assessed the influence of butyrate upon expression of cyclin-dependent kinase inhibitors *CDKN1A* (*p21*^*waf1/cip1*^) and *CDKN1B* (*p27*^*kip1*^), two key target genes whose promoters contain butyrate responsive elements and are known drivers of the HDACi effects of butyrate upon differentiation of colonocytes^66–68^; however, their expression was not augmented in butyrate-treated HEK (Fig. 6e). Overall, these data indicate butyrate does not drive HEK differentiation by eliciting HDACi. We and others have shown that butyrate can shape functionality of CD8^+^ T lymphocytes by altering cellular metabolism^26,33^. Thus, we next examined the metabolic profile of epidermal keratinocytes isolated from control or butyrate-treated mice using a multiple pathway targeted analysis. We found that butyrate supplementation led to profound alterations of the keratinocyte metabolome (Fig. S4A). Pathway topology and enrichment analyses of the 108 differentially expressed metabolites revealed pronounced changes in metabolic pathways linked to mitochondrial fatty acid β-oxidation (FAO) and FA synthesis (Fig. 6f). SCFA, and butyrate in particular, have been shown to influence cellular metabolism by fueling mitochondrial FAO^26,33,69^. We found that HEK express *SLC16A1*, a known butyrate transporter^70^ but not *SLC5A8* (Fig. S4B). To ascertain whether butyrate is metabolized after entering keratinocytes, we treated HEK with ^13^C-labeled butyrate and performed isotopic profiling on cell lysates. ^13^C was incorporated in several intermediates and products of the tricarboxylic acid (TCA) cycle, particularly citrate (Fig. 6g). These data indicate that butyrate enters HEK and is subsequently catabolized to acetyl-CoA by β-oxidation followed, at least partly, by incorporation in the TCA cycle, an event known to occur in colonocytes^69^. Concomitantly, FAO-generated acetyl-CoA can serve as substrate for *de novo* FA synthesis and subsequent elongation into LCFA and very long-chain fatty acids (VLCFA). We found that butyrate increased expression of central enzymes involved in FA biosynthesis (*ACC)* and elongation into LCFA and VLCFA (*ELOVL4*) (Fig. 6h). These in vitro findings are supported by in vivo data showing that naive and HDM allergen-sensitized skin from butyrate-treated animals contained increased LCFA and VLCFA (Fig. S4C). This phenomenon was restricted to the skin as systemic LCFA and VLCFA levels were largely unaltered by butyrate (Fig. S4D). Altogether, these results indicate that butyrate enhances keratinocyte-derived LCFA and VLCFA synthesis both in vitro and in vivo, a key event for subsequent generation of lipid species (e.g., ceramides) required for skin barrier function. We next tested whether butyrate-induced fueling of FAO and TCA cycle led to mitochondrial alterations. Flow cytometry analysis revealed that mitochondria from butyrate-treated HEK displayed increased mass and membrane potential (MMP), as determined by MitoTracker green probe incorporation and bivariate JC-1 dye red to green ratio, respectively (Fig. 6i). This augmented mitochondrial mass confirms TEM micrographs revealing enlarged mitochondrial networks in butyrate-treated HEK (Fig. 5i). These in vitro observations are further supported by in vivo data showing that already at baseline, keratinocytes from butyrate-exposed mice exhibited elevated MMP and that their mitochondrial mass increased with differentiation (Fig. S4E). These changes were further pronounced after allergen exposure (Fig. S4F), supporting the conclusion of an accelerated differentiation program in butyrate-supplemented keratinocytes following allergen-induced skin injury. To determine whether these morphological alterations had an impact on mitochondrial function, we measured oxygen consumption rates in HEK cultures. We found that mitochondria from butyrate-supplemented HEK displayed reduced basal respiration and markedly lowered maximal respiratory capacity (Fig. 6j), which did not translate into increased glycolysis as an alternative energy source (Fig. S4G). These data indicate that butyrate leads to a pronounced mitochondrial respiratory dysfunction and diminished fitness (Fig. S4H). Another important pathway utilized by butyrate to exert its effects on cellular metabolism is by enhancing LCFA intake^33^. Using a fluorescently-labeled synthetic FA we found that butyrate augmented the ability of HEK to incorporate LCFA (Fig. 6k). This finding was further supported by increased surface expression of *SLC27A1* and *SLC27A2* on butyrate-treated HEK, two essential transporters and activators of exogenous LCFA (Fig. 6l). Once acyl-CoA-activated, LCFA can serve as direct substrates for ceramide synthesis or be transported into the mitochondria via carnitine palmitoyltransferase (CPT)-1 to further fuel the FAO pathway. The fact that butyrate-exposed keratinocytes have a greater capacity to produce (Figs. 6h and S4C) as well as uptake LCFA (Fig. 6k, l) indicates that butyrate promotes mitochondrial β-oxidation in HEK not only by acting as a direct FAO substrate, but also indirectly via LCFA metabolism. To test this hypothesis, we directly quantified FAO in HEK. We found that butyrate augmented FAO activities (Fig. 6m), confirming a superior utilization of the FAO pathway in butyrate-exposed keratinocytes. To elucidate whether this enhanced FAO was responsible for increased HEK differentiation following butyrate supplementation, we used etomoxir, a pharmacological inhibitor that blocks β-oxidation. Etomoxir abrogated the effects of butyrate on HEK size, granularity, lysosomal contents, and mitochondrial mass (Fig. 6n). Altogether, these data indicate that butyrate promotes epidermal differentiation primarily by enhancing mitochondrial FAO in keratinocytes.

## Discussion

Over the past decade, dietary fibre and SCFA have received considerable attention with studies revealing their immunomodulatory capability to either suppress or enhance immune responses depending on the inflammatory context^25,26,33,71^. Comparatively, the impact of SCFA on structural cells remains poorly understood. We report here that dietary fibre and the SCFA, butyrate, reduce the magnitude of disease in a model of allergen-mediated ADLSI primarily by strengthening skin barrier function before, and particularly following allergen exposure. Indeed, our present study shows that butyrate acts on epidermal keratinocytes, altering their metabolism to promote and accelerate their differentiation. This resulted in enhanced skin barrier function, thereby impeding allergen penetration, allergic sensitization, and ultimately disease onset. Although our findings suggest that butyrate primarily functions to improve barrier function in this model, we acknowledge that not all immune cells present in the skin environment were investigated in our studies, and it is possible that butyrate affects immune cells secondary to its effect on keratinocytes. Of note, unlike others, we did not find that butyrate regulates skin inflammation by expanding Treg^29,72^. These discrepancies could be attributable to the fact that our model employs a repeated exposure of protease-active HDM allergens on the dorsal skin whilst these studies used models of ear contact hypersensitivity^73^. Numerous models have been developed to study AD-like disease in mice. Unlike other models that employ ovalbumin (allergen without skin barrier-degrading capacities) or tape-stripping (mechanical skin barrier disruption without allergen exposure), our model is based on protease-active HDM allergens which can naturally lead to both epidermal barrier dysfunction^74^ and subsequent allergen sensitization. It is noteworthy to underline that not only is the human skin continuously exposed to substantial amounts of HDM allergens (up to over a 100 µg per g of household dust, while our model relies only on 15 µg of HDM allergens per exposure)^75,76^, but they can also be detected directly on the skin^77^.

Sanford and colleagues reported that butyrate modulates inflammatory gene expression by inhibiting histone acetylation in primary human keratinocytes^65^. Our work shows that butyrate promotes keratinocyte differentiation, not by exerting HDACi effects, but rather by fueling FAO (directly and indirectly) and altering mitochondrial metabolism and functionality. This disparity could be attributable to the different concentrations used in both models. Sanford et al. used 2 mM butyrate while 0.5 mM was used in our studies. As described by Goncalves et al., similar mechanistic differences were observed in colonocytes where concentrations of butyrate below 1 to 2 mM drive FAO-mediated effects while concentrations above this range were associated with HDACi properties^70^. This dichotomic function can be explained by the oxidative capacity of colonocytes which was reported to be approximately between 1 and 2 mM^74^. Epidermal keratinocytes might possess a similar oxidative capacity to that of colonocytes, explaining why butyrate could mediate HDACi effects at high concentrations (mM range) but rather acts as an energy source fueling the FAO pathway at lower concentrations. Along similar lines, Kolly et al. reported that p21^cip1^ and p27^kip1^, two key target genes driving butyrate-mediated HDACi pro-differentiation effects on colon cells^66–68^, were implicated in the differentiation of murine keratinocytes^75^. The authors demonstrated that increased expression of these genes was triggered by a “confluency switch” and preceded terminal differentiation of keratinocytes. In our in vitro model, HEK were supplemented with butyrate before confluency, a possible explanation why, in our model, butyrate does not require p21^cip1^ and p27^kip1^ to elicit its pro-differentiating effects upon keratinocytes.

A further finding from our study is the ability of butyrate to alter lipid metabolism in epidermal keratinocytes, particularly following allergen-mediated skin injury. We show here that butyrate can promote lipid synthesis and the production of LCFA, VLCFA, cholesterol, and ceramides, all necessary for skin barrier function. This observation is supported by previous research showing that butyryl-CoA, which is obtained by conversion of butyrate via the enzyme butyryl-CoA synthase, is the most effective acyl-CoA compound in stimulating conversion of malonyl-CoA into LCFA^76^. This capacity of butyrate to shape lipid metabolism in epithelial cells could be of particular interest in the context of several lung diseases. Pulmonary surfactant, a critical lipoprotein complex exclusively produced by alveolar epithelial cells, is reduced in disorders such as chronic obstructive pulmonary disease, acute respiratory distress syndrome, and idiopathic pulmonary fibrosis. Our lipidomics analysis indicates that several of its constituents (e.g., cholesterol, phosphatidylcholine (data not shown)) are augmented in butyrate-exposed keratinocytes. Hence, future studies could assess the effect of butyrate supplementation upon pulmonary surfactant synthesis under homeostatic conditions and disease.

Recent studies have suggested SCFA-producing gut bacteria may influence atopic dermatitis and allergic sensitization in infants and young children^41–43^. Song et al. reported that the gut microbiota from children with AD–especially those under 1 year of age–is enriched in a subspecies *of Faecalibacterium prausnitzii*, specifically strain L2-6, a poor SCFA-producer. Fecal samples from children with AD contained lower levels of SCFA, especially butyrate. The authors showed that strain L2-6 replaced other strains such as strain A2-165, a high-butyrate producer, which was found in greater abundance in healthy control infants. The authors further demonstrated that strain L2-6 was the sole subspecies enzymatically equipped to rely on carbohydrates from endogenous mucins as its main nutrient source, rather than carbohydrates from dietary fibre. This finding suggests that the gut microbiome of AD infants has evolved as a consequence of environmental changes, and more specifically, deprivation of dietary fibre. An enrichment in mucus-degrading bacteria in low-fibre diet-exposed individuals results in greater epithelial access to potential barrier-damaging agents such as allergens and pathogens, a concept that has been experimentally supported^77^. Moreover, in light of our data on the cutaneous barrier, as well as work from others in the gut^36,37^, the fact that mucus-eroding bacteria are less efficient at producing SCFA, particularly butyrate, further puts the epithelial barrier at risk in dietary fibre-deprived individuals. Importantly, the findings from Song et al. are not restricted to AD. Indeed, a deficiency in the high-butyrate-producing strain A2-165 has also been implicated in patients with Crohn’s disease, a disorder that also results from epithelial barrier impairment^78,79^.

In summary, we show here that consumption of dietary fibre is pivotal for promoting skin barrier function, thereby restricting allergen ingress and subsequent sensitization and disease. Taken together, our work supports the concept that reduced SCFA levels, characteristic of low-fibre diet and a “westernized lifestyle”, impairs epithelial barrier function, and highlights the gut-skin axis as a central mechanism underpinning protection against allergies and atopic dermatitis.

## Experimental model and subject details

### Experimental animals

Three-week-old BALB/c female mice were purchased from Charles River Laboratories (L’Arbresle, France) and housed under specific pathogen-free conditions. All animal experiments were performed in accordance with institutional guidelines and Swiss federal and cantonal laws on animal protection.

### Rodent diets

Mice were fed a low-fibre diet (Granovit diet 2122) either supplemented with 30% inulin (fibrulin instant, Cosucra) or 30% cellulose (J. Rettenmaier & Söhne, Rosenberg, Germany) for 4 weeks before HDM allergen exposure and throughout the experiment. For SCFA-supplementation experiments, weanling mice were fed a low-fibre diet (Granovit diet 2122) without any additives for 2 weeks before epicutaneous allergen exposure and throughout the experiment. All diets were purchased from Granovit, Kaiseraugst, Switzerland.

### Short-chain fatty acid (SCFA) treatment

Weanling female mice received sodium acetate, propionate, or butyrate (Sigma-Aldrich, St. Louis, MO) in the drinking water at a final concentration of 400 mM for 2 weeks before epicutaneous allergen exposure and throughout the experiment. For baseline skin experiments, mice were fed low-fibre diet and administered butyrate as mentioned above but not exposed to allergen epicutaneously. For such experiments, skin of baseline animals was collected at the end of butyrate treatment.

### Animal model of allergen-induced skin barrier dysfunction and atopic dermatitis-like skin inflammation

ADLSI was induced experimentally by following an established murine model^44^. Briefly, adult (6 to 8 weeks old) female mice were anaesthetized with a mixture of ketamine and xylazine (Dr. E. Graeub AG, Bern, Switzerland). The dorsal skin was shaved and a gauze (Dermaplast, IVF Hartmann AG, Neuhausen, Switzerland) was instilled with 15 μg of house dust mite (HDM) extract from Dermatophagoides pteronyssinus^25,44^ (Stallergenes Greer, Lenoir, NC, part number XPB70D3A25, lot number 348717) diluted in 80 μL saline and thereafter applied onto the shaved skin. The compress was secured using a bioclusive dressing (Systagenix, Gatwick, UK) and further wrapped with an adhesive fabric (Mefix, Mölnlycke Health Care AB, Göteborg, Sweden) to prevent gauze removal and ingestion of HDM allergens. Epicutaneous sensitizations occurred for 1 or 2 consecutive weeks on Mondays and Wednesdays, depending on the experimental setting. Between 2 sensitizations (on Wednesdays), the gauze and dressing were gently removed and a fresh patch was applied onto the dorsal skin. Shaving was performed again if necessary. On the 5th day of sensitization of each week (i.e., Fridays), the gauze and dressing were removed to allow for a 2-day recovery period.

### Primary human epidermal keratinocyte cultures

Primary human epidermal keratinocytes (HEK) (LGC standards, Molsheim, France) were cultured in dermal cell basal medium supplemented with keratinocyte growth kit, penicillin-streptomycin-amphotericin B solution, and phenol red (all from LGC standards) according to manufacturer’s instructions. HEK were seeded at 3000 to 5000 cells per cm^2^ and exposed to vehicle control, butyrate, or valproate when cells reached 50% to 70% confluency. Butyrate was mixed into the culture medium at a concentration of 0 (vehicle control; CTL) or 500 μM final (Butyrate; BUT) for 24 h, 48 h, or 96 h depending on the experimental question. In experiments described in Fig. 6, HEK were also stimulated for 48 h with 500 μM valproate final (VPA) to assess the role of HDAC inhibition (HDACi) by SCFA in eliciting epidermal differentiation in our in vitro model. In experiments outlined in Fig. S5, Ca^++^ (Sigma-Aldrich) was added to the cell culture media at 1.2 mM final concentration to promote calcium-induced keratinocyte differentiation. In experiments described in Fig. 5, cells were exposed to 20 μM etomoxir (Sigma-Aldrich) to block fatty acid oxidation.

## Method details

### Measurement of trans-epidermal water loss

Trans-epidermal water loss (TEWL) was assessed using a TEWAmeter TM 300 (C.K. Electronic Gmbh, Koln, Germany). Measurements were performed on baseline skin or at the end of each week of allergen sensitization (i.e., Fridays), 2 h after the gauze and dressing were removed. The TEWAmeter probe was applied directly onto the area where the gauze was placed in order to directly measure water loss and therefore evaluate skin barrier integrity.

### Assessment of skin disease severity

Dorsal skin exposed to HDM allergen was assessed for the presence or absence of the following criteria: dryness, rash, excoriation, baldness, and lichenification. Each symptom was scored as 1 if present or 0 if absent. The final disease severity score which is shown in the figures represents the sum of these individual scores.

### Histology

Skin tissue from the HDM allergen-exposed area was collected and fixed in 10 mL of 10% buffered formalin for 24 h at 4 °C. The next day, formalin was washed off with PBS and skin tissue was stored at 4 °C in PBS until being paraffin-embedded. Prepared sections (4 μm or 6 μm, respectively) were stained with (i) Hematoxylin and Eosin (H&E) reagents using standardized protocols to visualize and quantify acanthosis, immune cell infiltration, and *stratum corneum* (SC) thickness; or (ii) with 1 μg ml^−1^ anti-mouse loricrin antibody (905104, Biolegend, San Diego, CA) to visualize and quantify loricrin expression by immunohistochemistry (IHC) technique. Sections were analysed using a Leica DM4B microscope equipped with a Leica DMC2900 camera (Leica GmbH, Germany) and Leica Application Suite X (LSX) software version 2.0.0.14332.2. Epidermal and SC thicknesses were quantified using 3 random measurements per picture with an average of 7–8 pictures per sample, while Loricrin expression was quantified using the staining from the whole picture with an average of 4 pictures per sample. Quantification of epidermal thickness, loricrin expression, and SC thickness were performed using ImageJ software (NIH, Bethesda, MD).

### Phase-contrast microscopy

Primary human epidermal keratinocytes (HEK) were analysed by phase-contrast microscopy for assessment of morphological alterations using EVOS^®^ FL cell imaging system (Thermo Fisher Scientific).

### Transmission electron microscopy

Vehicle or butyrate (500 μM)-stimulated primary human epidermal keratinocytes (HEK) were directly fixed in 100 mm cell culture dishes using a 2.5% glutaraldehyde solution (EMS, Hatfield, PA, US) in Phosphate Buffer (PB 0.1 M, pH 7.4) (Sigma-Aldrich, St Louis, MO) during 1 h at room temperature (RT). Cells were then directly post-fixed by a fresh mixture of 1% OsO_4_ (EMS, Hatfield, PA) with 1.5% of C_6_FeK_4_N_6_ (Sigma-Aldrich) in PB buffer during 1 h at RT. The samples were then washed three times in distilled H_2_O and spun down in low melting agarose (2% in H_2_O) (Sigma-Aldrich), allowed to solidify on ice, cut into 1 mm^3^ cubes, and dehydrated in C_3_H_6_O solution (Sigma-Aldrich) at graded concentrations (30–40 min; 50–40 min; 70–40 min; 100% −2 × 1 h). This was followed by infiltration in Epon (Sigma-Aldrich) at graded concentrations (Epon 1/3 C_3_H_6_O −2 h; Epon 3/1 C_3_H_6_O −2 h, Epon 1/1–4 h; Epon 1/1–12 h). Samples were finally polymerized for 48 h at 60 °C in the oven. Ultrathin sections of 50 nm were cut on a Leica Ultracut (Leica Mikrosysteme GmbH, *Vienna*, Austria) and picked up on a copper slot grid 2 × 1 mm (EMS, Hatfield, PA, US) coated with a polystyrene film (Sigma, St Louis, MO, US). Sections were poststained with 2% C_4_H_6_O_6_U (Sigma-Aldrich) in H_2_O for 10 min, rinsed several times with H_2_O followed by Reynolds lead citrate in H_2_O (Sigma-Aldrich) for 10 min, and rinsed several times with H_2_O. Micrographs were taken with a transmission electron microscope Philips CM100 (Thermo Fisher Scientific, Waltham, MA) at an acceleration voltage of 80 kV with a TVIPS TemCam-F416 digital camera (TVIPS GmbH, Gauting, Germany).

### Mitochondrial fatty acid oxidation (FAO)

Mitochondrial FAO activities were quantified in primary human epidermal keratinocytes (HEK) stimulated for 48 h with either vehicle control or 500 μM butyrate using a colorimetric FAO assay kit (AssayGenie, Dublin, Ireland). The assay was performed according to the manufacturer’s instructions and the colorimetric reaction was read at 492 nm on a Synergy H1 microplate reader using Gen5 software version 3.08 (BioTek, Luzern, Switzerland). FAO activities were normalized to cell number as well as to protein content as determined by Pierce^TM^ bicinchoninic acid (BCA) assay (Thermo Fisher Scientific).

### Quantitative polymerase chain reaction (qPCR)

Total RNA was isolated from FACS-purified mouse keratinocytes and human epidermal keratinocytes (HEK) using a TriReagent^®^ (MRC, Cincinnati, OH)/isopropanol phase separation method, followed by Reliaprep^TM^ RNA cleanup and concentration kit (Promega, Madison, WI). Total RNA from murine skin was isolated using Reliaprep^TM^ RNA Tissue Miniprep System (Promega) following the manufacturer’s instructions.

Relative *β-Actin, S100a8*, *Il-33*, *Il-20*, *Il-4Rα*, *Il-1β*, *Cxcl1*, *Areg, Sema3d, Keratin 1 (Krt1), Involucrin (Ivl), Loricrin, Filaggrin (Flg), Lce1m, Clsp, Elovl6, Cers3, Smpd1, β3Galt2*, and *Acc* mRNA expression in murine skin or in FACS-purified mouse keratinocytes was assessed by quantitative RT-PCR using the following primer sets: *S100a8* forward 5′-TTCGTGACAATGCCGTCTGA-3′ and reverse 5′-TTATATTCTGCACAAACTGAGGAC-3′; *Il-33* forward 5′-GCTGCGTCTGTTGACACATT-3′ and reverse 5′-CACCTGGTCTTGCTCTTGGT-3′; *Il-20* forward 5′-GGCTTTGGTCTTGCCTTTGG-3′ and reverse 5′-TCTTCAGCTTGCACACTATCC-3′; *Il-4Ra* forward 5′-TCTGCATCCCGTTGTTTTGC-3′ and reverse 5′-GCACCTGTGCATCCTGAATG-3′; *Il-1b* forward 5′-CCCAAGCAATACCCAAAGAAGAAG-3′ and reverse 5′-TGTCCTGACCACTGTTGTTTCC-3′; *Cxcl1* forward 5′-GCCTATCGCCAATGAGCTG-3′ and reverse 5′-ATTCTTGAGTGTGGCTATGA-3′; *Areg* forward 5′-AGCTGAGGACAATGCAGGGTA-3′ and reverse 5′-AGTGACAACTGGGCATCTGG-3′; *Sema3d* forward 5′-GTGTCCAAGCAAAACCTATGACC-3′ and reverse 5′-TGGGTACACGGACTTATACATCA-3′; *Krt1* forward 5′-TGGGAGATTTTCAGGAGGAGG-3′ and reverse 5′-GCCACACTCTTGGAGATGCTC-3′; *Ivl* forward 5′-CCCTCCTGTGAGTTTGTTTGGT-3′ and reverse 5′-TGTGGAGTTGGTTGCTTTGC-3′; *Loricrin* forward 5′-TCCCTCACTCATCTTCCCTG-3′ and reverse 5′-GGTCTTTCCACAACCCACAG-3′; *Flg* forward 5′-GTCTTCTTCCAAACAAGGTGCAT-3′ and reverse 5′-ACTGCCTGTAGTTGTCCTTCC-3′; *Lce1m* forward 5′-TCAATCCATTACTCGCTGAG-3′ and reverse 5′-GAAGAGGTTGGAGGGCACTT-3′; *Clsp* forward 5′-ACTAAGGAGGAGGTCGCTGA-3′ and reverse 5′-ATCTCCAAGTTCCTCGACGC-3′; *Elovl6* forward 5′-GAAAAGCAGTTCAACGAGAACG-3′ and reverse 5′-AGATGCCGACCACCAAAGATA-3′; *Cers3* forward 5′- TGAAGGTGCCATCCAGTAGC-3′ and reverse 5′-CGTTCCAGGCAGCTTTGTTC-3′; *Smpd1* forward 5′-TGGGACTCCTTTGGATGGG-3′ and reverse 5′-CGGCGCTATGGCACTGAAT-3′; *b3-Galt2* forward 5′-CCCTTACGGGTGTGCTTTCTC-3′ and reverse 5′-TGATAGCTCGGTGTTACTGGA-3′; *Acc* forward 5′-CGCTCACCAACAGTAAGGTGG-3′ and reverse 5′-GCTTGGCAGGGAGTTCCTC-3′; *β-Actin* forward 5′-GATCAAGATCATTGCTCCTCCTGA-3′ and reverse 5′-CAGCTCAGTAACAGTCCGCC-3′.

Relative β*-ACTIN*, *DSG1*, *KRT1*, *KRT10*, *IVL*, *FLG*, *CLSP*, *ACC, ELOVL4, SLC27A1, SLC27A2, HES1, HEY1, HEY2, CDKN1A*, and *CDKN1B* mRNA expression in primary human epidermal keratinocytes (HEK) was assessed by quantitative RT-PCR using the following primer sets:

*DSG1* forward 5′-AACCCAATCGCCAAAATTCACT-3′ and reverse 5′-ACCTCTCGATCAACTATGGATGT-3′; *KRT1* forward 5′-AGAGTGGACCAACTGAAGAGT-3′ and reverse 5′-ATTCTCTGCATTTGTCCGCTT-3′; *KRT10* forward 5′-TTGCTGAACAAAACCGCAAAG-3′ and reverse 5′-GCCAGTTGGGACTGTAGTTCT-3′; *IVL* forward 5′-GACTGTGTAAAGGGACTGCC-3′ and reverse 5′-CATTCCCAGTTGCTCATCTCTC-3′; *FLG* forward 5′-GGGAAGTTATCTTTTCCTGTC-3′ and reverse 5′-GATGTGCTAGCCCTGATGTTG-3′; *CLSP* forward 5′-GGTTGACACGGATGGAAACG-3′ and reverse 5′-ACTCCTGGAAGCTGATTTCGC-3′; *ACC* forward 5′-AGAAGACAAGAAGCAGGCAAAC-3′ and reverse 5′-GTAGACTCACGAGATGAGCCA-3′; *ELOVL4* forward 5′-AAGGACCGAGAACCTTTTCAGA-3′ and reverse 5′-TCCCGCATTATATGATCCCATGA-3′; *SLC27A1* forward 5′-GGGGCAGTGTCTCATCTATGG-3′ and reverse 5′-CCGATGTACTGAACCACCGT-3′; *SLC27A2* forward 5′-TACTCTTGCCTTGCGGACTAA-3′ and reverse 5′-CCGAAGCAGTTCACCGATATAC-3′; *HES1* forward 5′-CCTGTCATCCCCGTCTACAC-3′ and reverse 5′-CACATGGAGTCCGCCGTAA-3′; *HEY1* forward 5′-GTTCGGCTCTAGGTTCCATGT-3′ and reverse 5′-CGTCGGCGCTTCTCAATTATTC-3′; *HEY2* forward 5′-GCCCGCCCTTGTCAGTATC-3′ and reverse 5′-CCAGGGTCGGTAAGGTTTATTG-3′; *CDKN1A* forward 5′-TGTCCGTCAGAACCCATGC-3′ and reverse 5′-AAAGTCGAAGTTCCATCGCTC-3′; *CDKN1B* forward 5′-TAATTGGGGCTCCGGCTAACT-3′ and reverse 5′-TGCAGGTCGCTTCCTTATTCC-3′; *β-ACTIN* forward 5′-CCAACCGCGAGAAGATGA-3′ and reverse 5′-TCCATCACGATGCCAGTG-3′.

Ten microliter PCR reactions were set up containing 2 μL of template cDNA at a concentration of 50 ng ml^−1^, 5 μL SsoAdvanced SYBR Green reaction mix (Bio-Rad, Hercules, CA), 0.25 μL of each primer at a concentration of 20 μM, and 2.5 μL of nuclease-free H_2_O. Quantitative PCR was performed on the CFX96 Touch Real-Time PCR Detection System (Bio-Rad) using the following conditions: one cycle at 95 °C for 2 min and then 40 cycles at 95 °C for 15 s and 60 °C for 30 s, followed by a dissociation stage at 65 °C for 31 s and cycles of 5 s starting at 65 °C, raising 0.5 °C per cycle, to obtain melting curves for specificity analysis. Following amplification, C_q_ values were obtained using the CFX Maestro software 1.1 version 4.1.2433.1219 (Bio-Rad).

### Flow cytometry

Characterization of the main immune cell types, as well as that of epidermal keratinocytes in the murine skin, was performed by flow cytometry. Shaved dorsal skin tissue previously exposed to house dust mite allergen (lesional, ADLSI) or vehicle (baseline) was excised, weighed (for cell counts normalization purposes), and injected intradermally with 1 mL of a mixture of 0.5 mg ml^−1^ liberase TL (Roche, Mannheim, Germany) and 1 mg ml^−1^ DNase I (Roche) in RPMI 1640 medium (Thermo Fisher Scientific, Waltham, Ma) containing 55 μM β-mercaptoethanol (Thermo Fisher Scientific) and 20 mM HEPES (BioConcept AG, Allschwill, Switzerland). Skin tissue was thereafter incubated for 2 h at 37 °C, 5% CO_2_ with gentle agitation. Samples were then minced into small pieces using a razor blade and sequentially filtered through a 100 μm and 70 μm cell strainer using 10 mL of RPMI 1640 supplemented with 5% Fetal Calf Serum (FCS; Thermo Fisher Scientific). Following centrifugation, single-cell suspensions were washed and resuspended in PBS supplemented with 0.2% bovine serum albumin (BSA; Sigma-Aldrich, St Louis, Mo). Total cell numbers were determined using a Coulter Counter (IG Instrumenten-Gesellschaft AG, Basel, Switzerland). Skin immune cells were then stained for flow cytometry using the following antibodies and reagents: Zombie red fixable viability stain-PE-Texas red (423109, 1:2000 dilution in PBS), CD45-Fluorescein isothiocyanate (FITC) (109805, 1:800 dilution), I-A/I-E-Alexa Fluor (AF) 700 (107622, 1:600 dilution), CD64-Brilliant Violet (BV) 605 (139323, 1:400 dilution), CD11c-Allophycocyanin (APC)-Cy7 (117324, 1:600 dilution), CD11b-APC (101202, 1:600 dilution), Ly6c-Pacific Blue (PB) (128014, 1:1000 dilution), F4-80-BV650 (123149, 1:500 dilution), CD24-Phycoerythrin (PE)-Cy7 (101812, 1:800 dilution), CD103-Peridinin Chlorophyll Protein (PerCP)-Cy5.5 (121416, 1:400 dilution), Programmed Death-Ligand 2 (PD-L2)-PE (107205, 1:400 dilution), CD11b-PerCP-Cy5.5 (101228, 1:600 dilution), SiglecF-AF647 (BD Biosciences, 562680, 1:400 dilution), Ly6g-biotin (127604, 1:400 dilution), TCRγδ-biotin (118103, 1:200 dilution), Streptavidin-PE-Cy7 (405206, 1:800 dilution), CCR2-BV650 (BD Biosciences, 747968, 1:400 dilution), CD45-AF700 (109822, 1:400 dilution), CD3-PB (100214, 1:400 dilution), CD8-FITC (126606, 1:400 dilution), TCRβ-APC-Cy7 (109220, 1:200 dilution), CD44-PE (BD Biosciences, 553134, 1:400 dilution), FoxP3-AF647 (126408, 1:200 dilution), Lineage Cocktail-PB (133310, 1:50), CD90.1-FITC (BD Biosciences, 561973, 1:400 dilution), CD90.2-FITC (BD Biosciences, 553003, 1:400 dilution), CD45.2-FITC (109805, 1:800 dilution), ICOS-PerCP-Cy5.5 (313518, 1:200 dilution), CD25-PE-Cy7 (102016, 1:200 dilution), CX3CR1-PE (149005, 1:400 dilution), and CCR2-PE-Cy7 (150611, 1:400 dilution). IL-4, IL-5, and IL-17A intracellular expression in ILCs were assessed after 4 h incubation with Brefeldin A (Biolegend) and subsequent staining in a 0.5% solution of saponin from Quillaja bark (Sigma-Aldrich) and antibodies to IL-4-PE (504104, 1:200 dilution), IL-5-APC (504306, 1:100 dilution), and IL-17A-AF700 (506914, 1:200 dilution). IL-4, IL-5, and IL-17A production in CD4^+^ T cells was assessed after 4 h incubation with a mixture of 1X Brefeldin A (Biolegend), Phorbol Myristate Acetate (PMA; Sigma-Aldrich) (0.1 μM final), and ionomycin (Sigma-Aldrich) (1 μg ml^−1^ final), and subsequent staining in a 0.5% solution of saponin from Quillaja bark (Sigma-Aldrich) and antibodies to IL-4-PE (504104, 1:200 dilution), IL-5-APC (504306, 1:100 dilution), and IL-17A-AF700 (506914, 1:200 dilution).

Murine epidermal interfollicular keratinocytes were stained for flow cytometry or fluorescence-activated cell sorting (FACS) depending on the experimental purpose using the following set of antibodies and fluorescently-labelled probes: CD45-AF700 (109822, 1:400 dilution), CD34-PerCP-Cy5.5 (128607, 1:300 dilution), CD326-PE-Cy7 (eBioscience, 25-5791-80, 1:300 dilution), CD49f-PE (313611, 1:600 dilution), Calcein violet AM (425203, 10 nM in PBS). Mitochondrial alterations in murine keratinocytes were determined using staining with MitoTracker^®^ Green FM dye (Thermo Fisher Scientific; 100 nM final in PBS) for assessment of mitochondrial mass, and MitoTracker^®^ Red CMXRos dye (Thermo Fisher Scientific; 100 nM final in PBS) for assessment of mitochondrial membrane potential.

For the identification of progenitor cells, bone marrow was isolated from the mouse hind legs. To assess frequencies of macrophage-DC progenitors, bone marrow cells were stained with antibodies to Lineage Cocktail-PB (133310, 1:50), CD115-Alexa488 (135512, 1:200 dilution), c-kit-APC (105811, 1:400 dilution), CD11c-APC-Cy7 (117324, 1:600 dilution), and I-A/I-E–AF700 (107622, 1:800).

Flow cytometry-based characterization of primary human epidermal keratinocytes (HEK) was performed employing the following antibodies and probes: Calcein violet AM (425203, 10 nM in PBS), Zombie red fixable viability stain-PE-Texas red (423109, 1:2000 dilution in PBS), MitoTracker^®^ Green FM dye (Thermo Fisher Scientific; 100 nM final in PBS) for assessment of mitochondrial mass, MitoTracker^®^ Red CMXRos dye (Thermo Fisher Scientific; 100 nM final in PBS) and bivariate JC-1 dye (2 μM final; MedChemExpress, Sollentuna, Sweden) for assessment of mitochondrial membrane potential, BODIPY^TM^ FL C_16_-FITC (0.5 μM final; Thermo Fisher Scientific) for assessment of long-chain fatty acid uptake, and LysoTracker^TM^ deep red (50 nM final; Thermo Fisher Scientific) for quantification of acidic organelles such as lysosomes. If not indicated otherwise, antibodies were purchased from Biolegend (San Diego, CA). Fluorescence minus one (FMO) internal staining controls were set up by pooling cells from all the different experimental groups. Cells were acquired on BD Fortessa or LSR-II (BD Biosciences, San Jose, CA). Samples were analyzed using FlowJo 10.7.1 software (Tree Star Inc., Ashland, OR).

### Percutaneous allergen ingress

To assess allergen penetration into the skin of CTL or butyrate-treated mice, 100 μg FITC dextran (10 KDa, Sigma-Aldrich) diluted in saline (40 μL final volume) were topically applied onto the shaved dorsal skin before or after epicutaneous allergen sensitization. Fourteen hours later, skin was collected and the frequencies of FITC-positive cells were determined by flow cytometry using the antibodies listed previously. A negative control was included in each experiment by topically applying saline only to determine the background FITC fluorescence of each population of interest.

### Antigen uptake and processing

To assess antigen uptake and processing capacities of cutaneous antigen-presenting cells, 100 μg FITC-dextran (10 KDa, Sigma-Aldrich) or DQ^TM^-Ovalbumin (Thermo Fisher Scientific) diluted in saline (100 μL final volume) were administered intradermally into the shaved dorsal skin (unexposed or previously exposed to house dust mite allergen). Four hours later, skin was collected and the frequencies of FITC-positive cells were determined by flow cytometry using the antibodies listed previously. A negative control was included in each experiment by injecting saline only to determine the background FITC fluorescence of each population of interest.

### ELISA

Levels of circulating HDM allergen-specific IgE were assessed by ELISA. Briefly, 10 µg ml^−1^ of HDM extract (Stallergenes Greer, NC, USA) was prepared in carbonate buffer (pH 9.6) and coated overnight at 4 °C in NUNC Immuno MaxiSorp plates. After a 2-h blocking step in PBS supplemented with 1% BSA, serum samples were diluted in PBS and incubated overnight at 4 °C. The next day, alkaline phosphatase-conjugated goat anti-mouse IgE (Southern Biotech, AL, USA) was diluted by 1’000 in PBS supplemented with 0.2% BSA and added to the samples for 2 h at room temperature. After addition of 4-Nitrophenyl phosphate disodium salt hexahydrate (Sigma- Aldrich), the colorimetric reaction was read at 405 nm on the Synergy H1 microplate reader (Biotek, Luzern, Switzerland).

### RNA sequencing and data processing

Total RNA from skin was converted into cDNA libraries using the Illumina TruSeq RNA Sample Preparation Kit (Illumina) according to the manufacturer’s instructions. Libraries were submitted to high-throughput sequencing using Illumina HiSeq 2500 system (Genomic Technologies Facility, Lausanne, Switzerland). Sequencing data were processed using the Illumina Pipeline Software 1.82. Raw fastq files were processed using RNASik v1.4.7 with default settings^80^. RNASik wraps alignment, QC metrics, and quantification in the 1 script, giving a gene-annotated counts table for differential expression analysis. Alignment was performed using STAR v2.5.2 against the iGenomes (http://sapac.support.illumina.com/sequencing/sequencing_software/igenome.html). GRCm38 reference with the associated gene-model GTF. FeatureCounts^81^ quantified read counts to annotated genes. The data discussed in this manuscript have been deposited in NCBI’s Gene Expression Omnibus^82^ and are accessible through GEO Series accession number GSE185640 (https://www.ncbi.nlm.nih.gov/geo/query/acc.cgi?acc=GSE185640).

### RNA sequencing and gene ontology analysis

Visualization and analysis of differentially expressed genes were performed using Degust v4.1.5 (http://degust.erc.monash.edu/) and R statistical software version 4.1.0. Differentially expressed genes were visualized by volcano plot and heatmap linked to select gene ontology (GO) IDs as reported previously^44^ using R packages EnhancedVolcano version 1.10.0, alluvial version 0.1-2, and gplots version 3.1.1. GO enrichment analysis was performed using GOrilla (Gene Ontology enRIchment anaLysis and visuaLizAtion tool) (http://cbl-gorilla.cs.technion.ac.il). A list of all genes analysed in these samples was used as the background list in GOrilla. Differentially expressed genes were selected based on a false detection rate below 0.1 and a fold-change more than 2.

### Detection of ^13^C butyrate in skin tissue

To determine whether orally administered butyrate reaches the skin, mice were fed a low-fibre diet for 2 weeks, fasted overnight before being gavaged with a bolus of 0.8 mmole of butyrate-1-^13^C (Sigma-Aldrich, mass shift M + 1 isotope). Mice were sacrificed 45 min later; dorsal skin was collected and snap-frozen in liquid nitrogen before being stored at minus 80 °C until sample processing for isotopic profiling by Hydrophilic Interaction Liquid Chromatography coupled to high-resolution mass spectrometry (HILIC–HRMS).

### Metabolism of ^13^C butyrate in vitro

Primary human epidermal keratinocytes (HEK) were seeded in 100 mm cell culture dishes (Corning). When cells reached 60% confluency, HEK were stimulated with 500 μM butyrate-1^−13^C (Sigma-Aldrich, mass shift M + 1 isotope) for 24 h. HEK (roughly 10^6^ cells) were thereafter washed in PBS once and stored at minus 80 °C until processing for isotopic profiling using Hydrophilic Interaction Liquid Chromatography coupled to high-resolution mass spectrometry (HILIC–HRMS).

### ^13^C isotopic profiling (HILIC–HRMS)

Tissue samples (mouse skin) and primary human epidermal keratinocyte (HEK) lysates were pre-extracted and homogenized by the addition of 500 µL and 1000 µL of CH_3_OH:H_2_O (4:1), respectively in the air-cooled Cryolys Precellys Homogenizer (2 × 20 s at 10,000 rpm, Bertin Technologies, Rockville, US). Homogenized extracts were centrifuged for 15 min at 21,000 *g* and 4 °C, and the resulting supernatant was collected and evaporated to dryness in a vacuum concentrator (LabConco, Missouri, US). Dried sample extracts were re-suspended in CH_3_OH:H_2_O (4:1, v/v) before LC-HRMS analysis according to the total protein content (to normalize for sample amount). Sample extracts were analyzed by HILIC–HRMS in negative ionization mode using a 6550 Quadrupole Time-of-Flight (Q-TOF) system interfaced with a 1290 UHPLC system (Agilent Technologies). Raw LC-HRMS files were processed in Profinder B.08.00 software (Agilent Technologies) using the targeted data mining in isotopologue extraction mode. The metabolite identification was based on accurate mass and retention time matching against an *in-house* database containing data on 600 polar metabolite standards (analyzed in the same analytical conditions). The Extracted Ion Chromatogram areas (EICs) of each isotopologue (M + 0, M + 1, M + 2, M + 3,…) were corrected for natural isotope abundance^83^ and the label incorporation of ^13^C enrichment was calculated based on relative isotopologue abundance (in %), in each one of analyzed condition.

### Metabolite extraction and acquisition—multiple pathway targeted analysis

FACSorted murine interfollicular (CD326^+^ CD34^neg^) keratinocytes were pre-extracted and homogenized by the addition of 400 μL of CH_3_OH:H_2_O (4:1, v/v), in the Cryolys Precellys 24 sample Homogenizer (2 × 20 s at 10,000 rpm, Bertin Technologies, Rockville, MD, US) with ceramic beads. The bead beater was air-cooled down at a flow rate of 110 L min^−1^ at 6 bars. Homogenized extracts were centrifuged for 15 min at 4000 *g* at 4 °C (Hermle, Gosheim, Germany). The resulting supernatant was collected and evaporated to dryness in a vacuum concentrator (LabConco, Missouri, US). Dried sample extracts were resuspended in CH_3_OH: H_2_O (4:1, v/v) according to the total protein content for sample normalization. The protein pellets were evaporated and lysed in 20 mM Tris-HCl (pH 7.5), 4 M CH_6_ClN_3_, 150 mM NaCl, 1 mM Na_2_EDTA, 1 mM EGTA, 1% Triton, 2.5 mM Na_4_P_2_O_7_, 1 mM C_3_H_9_O_6_P, 1 mM Na_3_VO_4_, 1 mg ml^−1^ leupeptin using the Cryolys Precellys 24 sample Homogenizer (2 × 20 s at 10,000 rpm, Bertin Technologies, Rockville, MD, US) with ceramic beads. BCA Protein Assay Kit (Thermo Scientific, Massachusetts, US) was used to measure total protein concentration (Hidex, Turku, Finland). Extracted samples were analyzed by HILIC coupled to tandem mass spectrometry (HILIC–MS/MS) in both positive and negative ionization modes using a 6495 triple quadrupole system (QqQ) interfaced with a 1290 UHPLC system (Agilent Technologies). In positive mode, the chromatographic separation was carried out in an Acquity BEH Amide, 1.7 μm, 100 mm × 2.1 mm I.D. column (Waters, Massachusetts, US). The mobile phase was composed of A = 20 mM NH_4_HCO_2_ and 0.1% FA in H_2_O and B = 0.1% FA in ACN. The linear gradient elution from 95% B (0–1.5 min) down to 45% B was applied (1.5–17 min) and these conditions were held for 2 min. Then initial chromatographic conditions were maintained as a post-run during 5 min for column re-equilibration. The flow rate was 400 μl min^−1^, column temperature 25 °C, and sample injection volume 2 μl. ESI source conditions were set as follows: dry gas temperature 290 °C, nebulizer 35 psi and flow 14 L min^−1^, sheath gas temperature 350 °C and flow 12 L min^−1^, nozzle voltage 0 V, and capillary voltage 2000 V. Dynamic Multiple Reaction Monitoring (DMRM) was used as acquisition mode with a total cycle time of 600 ms. Optimized collision energies for each metabolite were applied. In negative mode, a SeQuant ZIC-pHILIC (100 mm, 2.1 mm I.D. and 5 μm particle size, Merck, Damstadt, Germany) column was used. The mobile phase was composed of A = 20 mM NH_4_CH_3_CO_2_ and 20 mM NH_4_OH in H_2_O at pH 9.7 and B = 100% ACN. The linear gradient elution from 90% (0–1.5 min) to 50% B (8–11 min) down to 45% B (12–15 min). Finally, the initial chromatographic conditions were established as a post-run during 9 min for column re-equilibration. The flow rate was 300 μl min^−1^, column temperature 30 °C, and sample injection volume 2 μl. ESI source conditions were set as follows: dry gas temperature 290 °C and flow 14 L min^−1^, sheath gas temperature 350 °C, nebulizer 45 psi, and flow 12 L min^−1^, nozzle voltage 0 V, and capillary voltage −2000 V. Dynamic Multiple Reaction Monitoring (dMRM) was used as acquisition mode with a total cycle time of 600 ms. Optimized collision energies for each metabolite were applied. Pooled QC samples (representative of the entire sample set) were analyzed periodically (every 5 samples) throughout the overall analytical run in order to assess the quality of the data, correct the signal intensity drift (attenuation in most cases, that is inherent to LC-MS technique and MS detector due to sample interaction with the instrument over time) and remove the peaks with poor reproducibility (CV > 30%). In addition, a series of diluted quality controls (QC) were prepared by dilution with CH_3_OH: 100% QC, 50% QC, 25% QC, 12.5% QC, and 6.25% QC. Metabolites were then selected also considering the linear response on the diluted QC series (R2 > 0.65). Raw LC-MS/MS data was processed using the Agilent Quantitative analysis software (version B.07.00, MassHunter Agilent technologies). Relative quantification of metabolites was based on EIC (Extracted Ion Chromatogram) areas for the monitored MRM transitions. The obtained tables (containing peak areas of detected metabolites across all samples) were exported to “R” software (http://cran.r-project.org/) and signal intensity drift correction and noise filtering (if necessary using CV (QC features) >30%) was done within the MRM PROBS software. Heatmap was generated using R package ComplexHeatmap version 2.8.0.

### Lipidomics analyses

Skin samples (40–50 mg) or human primary keratinocytes were homogenized in ice-cold chloroform/methanol (2:1, v/v), spiked with a mixture of deuterated internal standards (4 ng each of CER N(16)S(18)-*d*_*9*_, CER N(16)DS(18)-*d*_*9*_, CER N(16)H(18)-*d*_*9*_, CER N(16)P(18)-*d*_*9*_, CER A(16)S(18)-*d*_*9*_, CER A(16)DS(18)-*d*_*9*_, CER A(16)H(18)-*d*_*9*_, CER A(16)P(18)-*d*_*9*_, CER E(26)O(18:1)S(18)-*d*_*9*_ and CER E(26)O(18:1)P(18)-*d*_*9*_ (Cayman Chemicals, USA) and/or 8 µg of cholesterol-*d*_7_ (Avanti Polar Lipids, USA) and incubated in the dark. Next, ice-cold water was added and the organic layer was collected after centrifugation (5 min, 1500 *g*, 4 °C), evaporated under a N_2_ stream and the lipid residue reconstituted in 500 µL ice-cold chloroform. For the analysis of cholesterol, an aliquot (100 µL) of the lipid extract was collected and mixed with 100 µL of ice-cold isopropanol and kept at −20 °C until analysis by ultra-high performance supercritical fluid chromatography coupled to quadrupole-time-of-flight mass spectrometry with electrospray ionization (UHPSFC/ESI-QTOF-MS^E^). For the analysis of ceramides, the lipid extract (400 µL of skin lipid extract; 500 µL of cell lipid extract) was cleaned up by solid-phase extraction (SPE) using a 100 mg Silica-Si SPE cartridge (Strata SI-1 Silica, Phenomenex, UK). The SPE cartridge was conditioned with 2 mL ice-cold hexane before loading the 400 µL aliquot and then washed with 1.5 mL ice-cold chloroform. Ceramides were eluted with 2 mL ice-cold chloroform/methanol (2:1, v/v) and 2 mL ice-cold chloroform/methanol with 0.1% formic acid (2:1, v/v). The eluate was evaporated under a N_2_ stream, the lipid residue reconstituted in 200 µL methanol with 0.1% formic acid, and kept at −20 °C until analysis by ultrahigh performance liquid chromatography coupled to tandem mass spectrometry with ESI (UPLC/ESI-MS/MS). The analysis of cholesterol was performed by untargeted UHPSFC/ESI-QTOF-MS^E^ (UHPSFC, Acquity UPC^2^, Waters; Synapt G2 High Definition QTOF, Waters, Milford, MA, USA) in the positive ionization mode as detailed previously^84,85^. Chromatographic separation was achieved using an Acquity UPC^2^ Torus 2-PIC column (100 × 3 mm; 1.7 µm i.d.) (Waters, UK), CO_2_, and 30 mM ammonium acetate in methanol/water (99:1, v/v) as an organic modifier. Instrument control was carried out by MassLynx software (Waters, UK) and data processing by MassLynx and Progenesis QI software (NonLinear Dynamics, Newcastle, UK). Relative quantification was performed using deuterated internal standards.

The analysis of ceramides was carried out by UPLC/ESI-MS/MS (UPLC, Acquity, Waters; Xevo TQ-S, Waters, UK) as described previously^86^. Ceramides were separated on a C8 column (Acquity UPLC BEH C8; 100 × 2.1 mm; 1.7 μm; Waters, UK) under gradient elution using water and methanol both containing 0.1% formic acid. Detection was carried out by multiple reaction monitoring assays in the positive ionization mode. For data acquisition and instrument control and for integration and quantification MassLynx software and TargetLynx software (Waters, UK) were used, respectively. Ceramide identification was confirmed through retention times and characteristic fragmentation patterns^87^. Relative quantification was performed using deuterated internal standards. Protein content of the skin and cell samples was performed using a protein assay kit (Bio-Rad, Hemel Hempstead, UK) as described before^88^. Total tissue or cell fatty acids were analysed by gas chromatography coupled to flame ionization (GC-FID) as described before^89^. Briefly, blood or skin tissue samples were defrosted on ice, homogenized, and lipids extracted with chloroform: methanol (4 mL; 2:1, v/v) containing 0.01% (w/v) butylated hydroxytoluene. Fatty acids were converted to fatty acid methyl esters (FAME) via acid-catalyzed transesterification using boron trifluoride in methanol; heneicosanoic acid (21:0) was used as an internal standard. FAME were separated on a BPX70 capillary column (60 m length, 0.25 mm internal diameter, 0.25 μm film) (Phenomenex, UK) on an Agilent 6850 GC system and autosampler (Agilent technologies).

### Mitochondrial respiration and glycolysis assays

Oxygen Consumption Rate (OCR; in pmoles min^−1^) and ExtraCellular Acidification Rate (ECAR; in mpH min^−1^) were measured using a Seahorse XF-96 metabolic extracellular flux analyzer (Agilent). Primary human epidermal keratinocytes (HEK) were seeded onto pre-coated Seahorse XF96 V3-PS cell culture microplates (Agilent) at a density of 10^3^ cells per well and stimulated with vehicle control or 500 μM butyrate 2–3 days later when reaching 50–70% confluency. For Mito Stress Tests aiming at measuring OCR and assessing the mitochondrial respiration of cells, HEK were collected 48 h post-stimulation, washed twice, and incubated in serum-free dermal cell basal medium (LGC standards) supplemented with keratinocyte growth kit (LGC standards) and containing glucose, pyruvate, and glutamine as respiratory substrates. Respiratory chain inhibitors were added as indicated in Fig. 6 at the following concentrations: oligomycin, 2.5 μg ml^−1^; Carbonyl Cyanide 4-(trifluoromethoxy)phenylhydrazone (FCCP), 2 μM; rotenone, 1 μM; and antimycin A, 1 μg ml^−1^. Respiration rates were determined every 6 min according to the following protocol: 2 min mixture; 2 min wait; and 2 min measurements. All experiments were performed at 37 °C. The maximal respiratory capacity was calculated as the difference between the respiration after the addition of FCCP and the one after rotenone and antimycin A addition (in OCR). OCR values were normalized by protein content. For Glycolysis Stress Tests aiming at measuring ECAR and evaluating the glycolytic capacity of cells, HEK were collected 48 h post-stimulation, washed twice, and switched to a version of Krebs-Ringer bicarbonate HEPES (KRBH) buffer with low proton buffering capacity that contained 0 mM glucose, 140 mM NaCl, 3.6 mM KCl, 0.5 mM NaH_2_PO_4_, 0.5 mM MgSO_4_, 1.5 mM CaCl_2_, 0,5 mM HEPES, and 0,5 mM NaHCO_3_ (pH 7.4). The perturbation profiling was 10 mM Glucose, 2.5 μg ml^−1^ oligomycin; and 20 mM 2-Dx-Glucose (2-DG), as outlined in Fig. S6. Extracellular acidification rates were also determined every 6 min following the next protocol: 2 min mixture; 2 min wait; and 2 min measurement. All experiments were performed at 37 °C. The glycolytic capacity was calculated as the difference between the basal extracellular acidification rate before glucose addition and the maximal glycolytic rate after sequential addition of glucose and oligomycin (in ECAR). ECAR values were normalized by protein content. All chemical compounds were purchased from Sigma-Aldrich.

## Quantification and statistical analysis

Student’s *t-test* (unpaired, two-tailed) or Mann–Whitney test (in the case of a non-Gaussian distribution of the samples) were used to calculate significance levels between treatment groups. Statistical parameters are reported in each figure legend. n represents the number of biological replicates. All results are expressed as mean ± SEM where SEM represents the Standard Error of the Mean. *NS* = not significant. *P* < 0.05 was considered significant. *P < 0.05, **P ≤ 0.01, ***P ≤ 0.001, ****P ≤ 0.0001. Variances were similar between compared groups in all experiments. No randomization strategy was used before conducting animal experiments. Investigators were not blinded when performing the experiments or analyses. Graph generation and statistical analyses were performed using Prism software version 9.2.0 (GraphPad, La Jolla, CA).

## Supplementary information

Supplementary Figures
