# Supplementary material for: Gut-derived short-chain fatty acids modulate skin barrier integrity by promoting keratinocyte metabolism and differentiation
Source: Mucosal Immunol. 2022 Jun 7;15(5):908–26. doi: 10.1038/s41385-022-00524-9 (PMC9385498; doi:10.1038/s41385-022-00524-9)
Supplement: Supplementary file 1 — Supplementary Figures [file 41385_2022_524_MOESM1_ESM.docx]

**Gut-derived short-chain fatty acids modulate skin barrier integrity by promoting keratinocyte metabolism and differentiation**

Aurélien Trompette^1,*^, MSc, Julie Pernot^1^, Olaf Perdijk^2^, PhD, Rayed Ali A. Alqahtani^3^, MSc, Jaime Santo Domingo^4^, PhD, Dolores Camacho-Muñoz^3^, PhD, Nicholas C. Wong^5^, PhD, Alexandra C. Kendall^3^, PhD, Andreas Wiederkehr^4^, PhD, Laurent P. Nicod^6^, MD, Anna Nicolaou^3^, PhD, Christophe von Garnier^1^, MD, Niki D.J. Ubags^1^, PhD, & Benjamin J. Marsland^2^, PhD.

**Affiliations:**

^1^Division of Pulmonary Medicine, Department of Medicine, Lausanne University Hospital (CHUV), University of Lausanne (UNIL), Lausanne, Switzerland

^2^Department of Immunology and Pathology, Central Clinical School, Monash University, Melbourne, Australia

^3^Laboratory for Lipidomics and Lipid Biology, University of Manchester, Division of Pharmacy and Optometry, Faculty of Biology Medicine and Health, Manchester Academic Health Science Centre, The University of Manchester, Manchester M13 9PT, UK

^4^Nestlé Institute of Health, EPFL innovation Park, Lausanne, Switzerland

^5^Monash Bioinformatics Platform, Monash University, Clayton, Victoria, Australia.

^6^Pneumologie, Clinic Cecil from Hirslanden, Lausanne, Switzerland

*Correspondence: [aurelien.trompette@chuv.ch](mailto:aurelien.trompette@chuv.ch)

**Figure S1. HFD and butyrate ameliorate ADLSI. Oral butyrate reaches skin. Related to Figure 1.** (**A**) Representative photographs of allergen-induced atopic dermatitis following allergen sensitization (four times 15 μg HDM over two weeks) onto the dorsal skin of control (cellulose; CD) or high-fiber diet (inulin; HFD) fed mice. (**B**) Representative photographs of allergen-induced atopic dermatitis following allergen sensitization (four times 15 μg HDM over two weeks) onto the dorsal skin of control (water control; CTL) or SCFA (butyrate)-orally supplemented mice. (**C**) Detection of ^13^C enrichment in the skin 45 minutes after oral supplementation with butyrate-1-^13^C (detected as mass shift “M+1” isotopologue) as assessed by hydrophilic interaction liquid chromatography coupled to high resolution mass spectrometry (HILIC - HRMS). Results are representative of data generated in three independent experiments in **A and B**, and one experiment in **C**. Results are expressed as mean ± SEM (*n* = 5 in **C**).

**Figure S2. Butyrate does not alter the skin immune environment before allergen exposure, nor after intradermal instillations of HDM allergens. Related to Figure 3.** (**A**) Main dendritic cell (DC) subsets (CD11b^+^ cDC2, Langherans cells (LCs), and Ly6c^neg^ mono-DCs) and their surface expression of Th2 activation marker program death-ligand 2 (PD-L2) in the skin of water control (CTL) or butyrate-treated animals before HDM allergen sensitization, as determined by flow cytometry. MFI, mean fluorescence intensity. (**B**) MHCII^+^ CD64^+^ CCR2^neg^ macrophages (the most prominent skin macrophage population in our model) and their expression of PD-L2 before allergen challenge, as determined by flow cytometry. (**C**) Skin γδT cells and their surface expression of activation marker CD44 in water control (CTL) or butyrate-treated mice before HDM allergen sensitization. (**D**) Total and IL-4-producing innate lymphoid cells (ILCs) in the skin, and their expression of IL-4 before HDM allergen challenges. (**E**) CD4^+^ T lymphocytes in the skin and their surface expression of CD44 in water control (CTL) or butyrate-supplemented animals before HDM allergen sensitizations. (**F**) Foxp3^+^ CD4^+^ T regulatory cells (Tregs) in the skin before HDM allergen challenges. (**G**) Main DC subsets (CD11b^+^ cDC2, LCs, and Ly6c^neg^ mono-DCs) and their surface expression of PD-L2 in the skin of water control (CTL) or butyrate-treated animals after two intradermal HDM allergen administrations (over one week), as determined by flow cytometry. (**H**) MHCII^+^ CD64^+^ CCR2^neg^ macrophages (the most prominent skin macrophage population in our model) and their expression of PD-L2 after two intradermal HDM allergen administrations, as determined by flow cytometry. (**I**) Skin γδT cells and their surface expression of activation marker CD44 after two intradermal HDM allergen administrations, as determined by flow cytometry. (**J**) Total ILCs in the skin and their expression of IL-4 after two intradermal HDM allergen administrations, as determined by flow cytometry. (**K-L**) CD4^+^ T lymphocytes in the skin and their surface expression of CD44 (**K**), as well as frequencies of IL-4-, IL-5, and IL-17A-producing CD4^+^ T cells (**L**) after two intradermal HDM allergen administrations, as determined by flow cytometry. (**M**) Foxp3^+^ CD4^+^ Tregs in the skin after two intradermal HDM allergen administrations, as determined by flow cytometry. Results are representative of data generated from three independent experiments in **A-B**, and from two independent experiments in **C-M**. All results are expressed as mean ± SEM (*n* = 6 per group in **A**, **B**, and **G-M,** and *n* = 5 per group in **C-F**). Statistical significance was determined with Student's *t*-test (unpaired, two-tailed) or a Mann-Whitney test (if samples did not follow a Gaussian distribution) in **A**-**M**.

**Figure S3. Butyrate supplementation potentiates calcium-induced differentiation of primary human keratinocytes *in vitro*. Related to Figure 5.** (**A**) Gene expression of several key markers of keratinocyte differentiation by primary human epidermal keratinocyte (HEK) cultures treated with vehicle (CTL) or butyrate (500 μM) for 48 hr in low (0.06 mM) or high (1.2 mM) Ca^++^ conditions, as determined by quantitative RT-PCR. DSG1, Desmoglein-1; KRT1, Keratin-1; IVL, Involucrin; FLG, Filaggrin. Gene expression was normalized to *β-ACTIN*. Results are representative of data from two to three independent experiments. All results are expressed as mean ± SEM (*n* = 2 per group). Statistical significance was determined with Student's *t*-test (unpaired, two-tailed) or a Mann-Whitney test (if samples did not follow a Gaussian distribution). *NS* = non-significant, **P* = 0.05 and ***P* = 0.01.

**Figure S4. Butyrate alters the metabolome of murine epidermal keratinocytes. Skin from butyrate-treated mice contain elevated levels of long-chain and very long-chain fatty acids. Epidermal keratinocytes from butyrate-treated mice show increased mitochondrial potential and mass. Butyrate impairs glycolytic function and capacity of primary human keratinocytes. Related to Figure 6.** (**A**) Heatmap depicting mean values obtained from a multiple pathway targeted metabolomic analysis of FACS-purified epidermal CD326^+^ keratinocytes from water control (CTL) or butyrate-treated mice after two house dust mite (HDM) allergen sensitizations, as determined by hydrophilic interaction liquid chromatography coupled to tandem mass spectrometry (HILIC - MS/MS). Colors used in the heatmap are not representative of the actual values but are indicative of whether values were lower or higher between the two groups. (**B**) Expression of butyrate transporters *SLC5A8* and *SLC16A1* by HEK cultures treated with vehicle (CTL) or butyrate for 48 hr, as assessed by PCR. (**C**) Levels of saturated long-chain and very long-chain fatty acids in baseline and HDM allergen-sensitized (“ADLSI”) skin of CTL or butyrate-exposed mice, as determined by gas chromatography with flame ionization (GC-FID). (**D**) Systemic levels of saturated long-chain and very long-chain fatty acids in red blood cells of naive (“baseline”) and HDM-sensitized (“ADLSI”) CTL or butyrate-treated mice, as determined by GC-FID. (**E**) Determination of mitochondrial membrane potential (MMP) (ratio of MitoTracker CMXRos expression (MFI, mean fluorescence intensity) to MitoTracker green MFI expression) and mitochondrial mass (MitoTracker green MFI) change during differentiation (CD49f^neg^ Vs. CD49f^+^) in CD326^+^ epidermal keratinocytes from CTL or butyrate-treated mice before HDM allergen sensitization, as determined by flow cytometry. (**F**) Determination of mitochondrial membrane potential (MMP) (ratio of MitoTracker CMXRos MFI expression to MitoTracker green MFI expression) and mitochondrial mass (MitoTracker green MFI) change during differentiation (CD49f^neg^ Vs. CD49f^+^) in CD326^+^ epidermal keratinocytes from CTL or butyrate-treated mice after two HDM allergen sensitizations, as determined by flow cytometry. (**G**) Glycolytic function and capacity of HEK either vehicle (CTL) or butyrate-treated for 48 hr, as determined by Seahorse glycolysis stress test assays. 2-DG = 2-Deoxy-D-Glucose. (**H**) Mitochondrial fitness of HEK either vehicle (CTL) or butyrate-treated for 48 hr, as determined by oxygen consumption rate (OCR) and extracellular acidification rate (ECAR) Seahorse assays. Results are representative of data generated in at least 3 independent experiments in **I**, from one experiment in **A-D**, from two independent experiments in **E**, and from three independent experiments in **F, G** and **H**. All results are expressed as mean ± SEM (*n* = 5 per group in **A, C, D, and F**; *n* = 6 per group in **E**; *n* = 8 per group in **G;** *n* = 13 in control group and *n* = 14 in butyrate group in **H**). Statistical significance was determined with Student's *t*-test (unpaired, two-tailed) or a Mann-Whitney test (if samples did not follow a Gaussian distribution) in **C**-**H**. *NS* = non-significant, **P* = 0.05, ***P* = 0.01, ****P* = 0.001, and *****P* = 0.0001.
